# An assessment of PCV13 vaccine coverage using a repeated cross-sectional household survey in Malawi

**DOI:** 10.12688/gatesopenres.12837.1

**Published:** 2018-08-02

**Authors:** Austin Bondo, Bejoy Nambiar, Norman Lufesi, Rashid Deula, Carina King, Gibson Masache, Charles Makwenda, Anthony Costello, Dale Rhoda, Eric D. McCollum, Tim Colbourn

**Affiliations:** 1 Parent and Child Health Initiative (PACHI) Trust, Lilongwe, Malawi; 2 UCL Institute for Global Health, London, WC1N 1EH, UK; 3 Acute Respiratory Infections Unit, Ministry of Health, Lilongwe, Malawi; 4 Biostat Global Consulting, Worthington, OH, 43085, USA; 5 Department of Pediatrics, Eudowood Division of Pediatric Respiratory Sciences, Johns Hopkins School of Medicine, Baltimore, USA; 6 Department of International Health, Johns Hopkins Bloomberg School of Public Health, Baltimore, USA

**Keywords:** 13-valent pneumococcal conjugate vaccine, PCV13, vaccine coverage, vaccination coverage survey, children, Malawi

## Abstract

**Background: **The 13-valent pneumococcal conjugate vaccine (PCV13) was introduced in Malawi from November 2011 using a three dose primary series at 6, 10, and 14 weeks of age to reduce *Streptococcus pneumoniae*-related diseases. To date, PCV13 paediatric coverage in Malawi has not been rigorously assessed.  We used household surveys to longitudinally track paediatric PCV13 coverage in rural Malawi.

**Methods: **Samples of 60 randomly selected children (30 infants aged 6 weeks to 4 months and 30 aged 4-16 months) were sought in each of 20 village clinic catchment ‘basins’ of Kabudula health area, Lilongwe, Malawi between March 2012 and June 2014. Child health information was reviewed and mothers interviewed to determine each child’s PCV13 dose status and vaccine timing. The survey was completed six times in 4-8 month intervals. Survey inference was used to assess PCV13 dose coverage in each basin for each age group. All 20 basins were pooled to assess area-wide vaccination coverage over time, by age in months, and adherence to the vaccination schedule.

**Results: **We surveyed a total of 8,562 children in six surveys; 82% were in the older age group. Overall, in age-eligible children, two-dose and three-dose coverage increased from 30% to 85% and 10% to 86%, respectively, between March 2012 and June 2014.  PCV13 coverage was higher in the older age group in all surveys. Although it varied by basin, PCV13 coverage was consistently delayed: median ages at first, second and third doses were 9, 15 and 21 weeks, respectively.

**Conclusion: **In our rural study area, PCV13 introduction did not meet the Malawi Ministry of Health one-year three-dose 90% coverage target, but after 2 years reached levels likely to reduce the prevalence of both invasive and non-invasive paediatric pneumococcal diseases. Better adherence to the PCV13 schedule may reduce pneumococcal disease in younger Malawian children.

## List of abbreviations

ARI        acute respiratory infection

IPD        invasive pneumococcal disease

IQR        inter-quartile range

LCB        lower 1-sided 95% confidence bound

LQAS     lot quality assurance sampling

PCV       pneumococcal conjugate vaccine

PCV13   13-valent pneumococcal conjugate vaccine

WHO      World Health Organisation

## Introduction

Acute respiratory infection (ARI) is a leading cause of death in children aged 0–59 months old worldwide. Pneumonia, a severe form of acute lower respiratory infection that affects the lungs, claims around 0.9 million lives of infants and young children annually ^1^. Pneumonia is more common in developing countries, specifically those in Africa and South Asia ^2, 3^. *Streptococcus pneumoniae* and *Haemophilus influenzae* are the most common causative agents of bacterial pneumonia in children and are targeted by the Pneumococcal conjugate vaccine (PCV) and the *H. influenzae* B vaccine, respectively ^2, 3^. PCV also prevents invasive pneumococcal disease (IPD) ^4^ and pneumococcal meningitis ^5^. In 2007, the World Health Organisation (WHO) recommended PCV be added to all national immunization programmes, especially in countries with high child mortality ^3^.

Malawi recorded around 500 ARI cases per 1000 under-5-years population between July 2009 and June 2010, of which approximately 1.5% died, according to health management information system statistics ^6^; the death rate is likely to be an under-estimate, as it is based on health facility data only. In November 2011, Malawi was among the first countries in Africa to introduce the 13-valent Pneumococcal Conjugate Vaccine (PCV13) ^7^ into its routine immunization program, aiming to protect millions of children from pneumococcal pneumonia and IPD. The vaccine was introduced as a three-dose schedule at 6, 10 and 14 weeks, with an initial catch-up campaign targeting all children up to the age of 1 year. A target national coverage rate of ≥90% and local district targets of ≥80% were set for each year from 2012 to 2016 ^8^.

We used a series of household surveys to assess changes in vaccine coverage in a rural area of central Malawi between March 2012 and June 2014. The surveys were part of a wider study exploring changes in the health-system burden of pneumonia following PCV13 vaccination introduction in two areas of central Malawi ^9^. We used the surveys to estimate local and area-wide vaccination coverage in one of our study areas to assess the burden of pneumonia at the community health worker, health centre and hospital levels in relation to the percentage of infants vaccinated. We aimed to quantify how the burden of pneumonia on the health system changed as vaccination coverage increased, and to track the roll-out of the PCV13 vaccine in Malawi in terms of the timeliness of each of the three doses in relation to the 6, 10, 14-week schedule and population coverage of each dose over time. In this paper we present the results of six surveys conducted in Lilongwe district.

## Methods

### Setting

This study took place in Kabudula, one of six health areas in Lilongwe district of the central region of Malawi, a low-income country in Sub-Saharan Africa with a population of approximately 18 million in 2016 and a GDP per capita of $1169 (purchasing power parity international dollars, 2016) ^10^. Lilongwe district is socio-economically reflective of Malawi, with youth female literacy of 72%, and net secondary school attendance of 16% ^11^. Under-5 mortality is rapidly improving in Malawi and was estimated to be 71 deaths per 1000 livebirths nationally in 2013, while this study was on-going ^12^.

### Sample size and data collection

We aimed to randomly sample 30 infants under 4 months and 30 infants aged 4–16 months in each of six waves of the survey in each of 20 village clinic catchment ‘basins’ in Kabudula. The target of 30 infants in each age group was chosen based on a lot quality assurance sampling (LQAS) decision rule of a cut-off of 19/30 children being unvaccinated (zero doses) yielding optimal power to determine whether the basin has above 50% PCV13 single dose coverage (with 95% power) or below 25% (with 89% power) ^13^ From the survey standpoint, the clinics are strata. Vaccination coverage can be calculated in each and then the data may be pooled to calculate an overall Kabudula-wide estimate. The village clinics are those in Kabadula that took part in the community component of the parent PCV13 vaccine study ^9^. We sought to estimate coverage for under-4 month-old and 4–16 month-old infants, in order to study the age-related dynamics of pneumococcal disease burden with respect to vaccine coverage. Measuring the infants age also enabled us to determine how closely the 6, 10, 14 week schedule was being followed. Timely vaccination was defined as per the standard WHO schedule: the first dose being received between 4 weeks and 2 months of age, the second dose between 8 weeks and 4 months and the third dose between 12 weeks and 6 months ^14^.

Six separate surveys were conducted in March 2012, October 2012, June 2013, October 2013, February 2014 and June 2014 following the introduction of the vaccine in November 2011.

Surveys were conducted by 12 experienced data collectors and a supervisor. Prior to each survey, data collectors were given a one-day intensive training on data collection, covering research ethics and etiquette, interviewing techniques, and means of verification. Data collection for each survey lasted 10 days.

**Figure 1.**
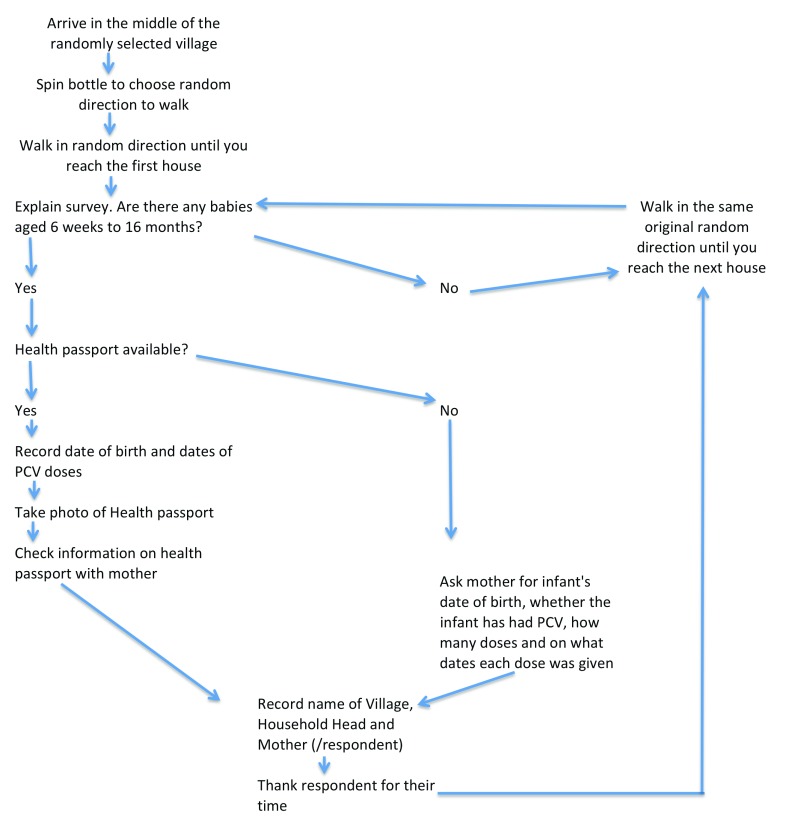
Data collection flow chart.

During the data collection exercise, a random walk method was employed to identify eligible infants for the survey. After arriving at the centre of each village, data collectors walked in a direction indicated by spinning a bottle, calling at every house on the way. At each house the data collector briefly explained that they were conducting a survey to find out how many infants had been vaccinated with PCV13. Then, they asked whether there were any infants aged between 6 weeks (old enough to have a vaccination) and 16 months (enough time for a third dose to be given to a child who was under age 1 year at first dose, the initial ‘catch-up’ target age group for the vaccination campaign). For respondents with infants, the data collector requested to see the infant’s health passport (a government provided health record) and it was inspected for vaccination records and date of birth. Old health passport books did not have a recording space for PCV13; in such cases the data collector inspected all pages to make sure that they did not miss any recorded vaccination data. A digital photograph or scan was taken of the page where the vaccination records were recorded. All data from the paper forms was single-entered into a computer for analysis and the digital photographs (available for 96% of records) were used to verify the entered data. In the event that the household did not have an eligible infant for the study, the data collectors moved to the next house and repeated the process. In cases where the infant did not have a health passport, data collectors relied on verbal information from the caregivers ( Figure 1). Data collection for the under-4-month-old infants often stopped before the target of 30 was reached in the village clinic basin due to no more infants of this age group being found. Additional infants in the older age group were sometimes surveyed to compensate. Both of these practices resulted in target sample sizes being missed for each age group for most village basins.

### Analysis

Due to considerable variation in the sample sizes obtained and our surveys often capturing a large proportion of the eligible population of children in each catchment area for each age-group (12–100%, median 47%), to simplify the analysis whilst ensuring informative results, we opted to analyse our data using survey-based inference rather than LQAS methods. Using a finite population correction that incorporated the total estimated eligible population, the number of infants surveyed and the number of infants vaccinated (either with one, two or three doses of PCV13) in each catchment area age-group sample, we calculated one-sided modified Wilson 95% confidence intervals (using the lower 95% confidence bound) ^15^ for the proportion vaccinated to estimate whether each age-group in each basin was above or below set target threshold levels of coverage of each dose of PCV13 ^16^. We use inchworm plots ^16^ to clearly visualise the confidence intervals in relation to the targets. If the lower one-sided 95% confidence bound (LCB) was above the target we infer that the target was achieved.

Overall percentage coverage of each PCV13 dose in each age group and by age in months for all 20 clinic catchment areas combined was also calculated for each of the six sequential surveys and trends over time were visualised and quantified. Our estimates of coverage of each dose are for age-eligible children, i.e. the denominator is all children at least 6 weeks (42 days) old for the first dose, all children at least 10 weeks (70 days) old for the second dose, and all children at least 14 weeks (98 days) old for the third dose. The data processing and graphs for these analyses were produced using Stata 13.1 and Stata 14.2 for Mac.

### Ethical approval and consent

Ethical approval was granted for this study by the National Health Sciences Research Committee in Malawi (reference: 941), and included approval for verbal consent to collect vaccination data from routine records (health passports). Verbal consent was given from all survey respondents who took part in this study, and was documented by the fieldworkers on the data collection form during data collection. The study was explained to respondents before verbal consent was given and verbal consent was only deemed necessary rather than written consent given low levels of literacy in the population and the fact the study was not asking about sensitive information. This study involved collection of vaccination data on infants under 16 months old—parental consent was given for this data to be collected as this data was collected from the parents themselves. This paper does not present any individual or identifiable patient data, therefore consent for publication is not applicable.

## Results

Table 1 details the overall findings for age and coverage of vaccination from all surveys. A total of 8,562 infants were recruited. Most infants (78–83%) were aged 4–16 months old at each time point. Overall 96.3% of the infants had health passports. The three-dose coverage of PCV13 vaccine in age-eligible children increased from 10.0% in March 2012 to 86.0% in June 2014. Similarly, there was also an increase during the same time period of the coverage of PCV13 doses 1 and 2 in age-eligible children from 62.0% and 30.3% respectively in March 2012 to 93.9% and 85.2% by June 2014. In all six surveys older infants (aged 4–16 months) were much more likely to be vaccinated (with one, two or three PCV13 doses) than infants aged less than 4 months old. Vaccination timeliness for the first, second and third PCV13 doses increased from 11%, 9% and 5% in March 2012 to 47%, 56% and 48% in June 2013 and 49%, 61% and 62% in June 2014 ( Table 1). The median ages at first, second and third doses mirrored this improvement, dropping throughout the six surveys and rapidly so in the first three surveys; overall they were 9 weeks (interquartile range (IQR), 7–13 weeks), 15 weeks (IQR, 12–20 weeks), and 21 weeks (IQR, 18–27 weeks; Table 1).

**Table 1.** Overall results of each and all surveys.

Variable		Wave						Total
		1 Mar'12	2 Oct'12	3 Jun'13	4 Oct'13	5 Feb'14	6 Jun'14	
Infants, N		1317	1393	1389	1498	1404	1561	8562
Health passports, %	No	7.6%	3.4%	2.7%	4.1%	2.4%	2.2%	3.7%
	Yes	92.4%	96.6%	97.3%	95.9%	97.6%	97.8%	96.3%
Age, %	<4 months	22.1%	16.2%	18.1%	18.0%	17.6%	17.1%	18.1%
	4–16 months	77.9%	83.8%	81.9%	82.0%	82.4%	82.9%	81.9%
Vaccination coverage, % (n age-eligible respondents ^) *	No doses	38.0% (1306)	9.4% (1391)	9.5% (1388)	8.3% (1488)	7.5% (1403)	6.1% (1561)	
	1 dose	62.0% (1306)	90.6% (1391)	90.5% (1388)	91.7% (1488)	92.5% (1403)	93.9% (1561)	
	2 doses	30.3% (1215)	81.2% (1313)	85.0% (1312)	83.6% (1407)	86.0% (1324)	85.2% (1491)	
	3 doses	10.0% (1113)	64.3% (1240)	72.3% (1235)	79.5 (1307)%	82.1% (1233)	86.0% (1384)	
Vaccination coverage * (n, total respondents &)	No doses	39.0%	9.0%	9.5%	8.4%	7.7%	6.0%	
	1 dose	61.0%	91.0%	90.5%	91.6%	92.3%	94.0%	
	2 doses	27.3%	76.9%	80.3%	78.7%	81.2%	82.2%	
	3 doses	8.5%	57.7%	64.3%	69.8%	72.4%	76.7%	
Vaccination coverage [<4 months] *, % (n age- eligible respondents ^) *	No doses	43.0% (281)	31.2% (223)	33.2% (250)	29.8% (269)	29.7% (246)	20.6% (267)	
	1 or more doses	56.0% (281)	70.8% (223)	66.8% (250)	70.2% (269)	70.3% (246)	79.4% (267)	
	2 or more doses	19.2% (190)	40.3% (145)	43.1% (174)	32.9% (178)	40.5% (167)	39.9% (197)	
	3 or more doses	8.6% (88)	8.4% (75)	8.2% (97)	9.4% (78)	16.3% (76)	29.8% (90)	
Vaccination coverage at 4–16 months, % (n total respondents) *	No doses	36.7% (1025)	5.1% (1168)	4.3% (1138)	3.5% (1229)	2.6% (1157)	2.9% (1294)	
	1 or more doses	63.3% (1025)	94.9% (1168)	95.7% (1138)	96.5% (1229)	97.4% (1157)	97.1% (1294)	
	2 or more doses	31.8% (1025)	86.8% (1168)	91.3% (1138)	91.1% (1229)	92.8% (1157)	92.7% (1294)	
	3 or more doses	10.1% (1025)	68.2% (1165)	77.7% (1138)	84.4% (1229)	87.0% (1157)	90.4% (1294)	
Timeliness, %	Timely 1 ^st^ dose †	11.1%	28.6%	46.7%	45.9%	46.3%	48.9%	38.5%
	Timely 2 ^nd^ dose ¶	8.5%	33.3%	55.8%	53.7%	55.9%	61.2%	45.5%
	Timely 3 ^rd^ dose §	4.9%	29.5%	47.7%	51.1%	53.1%	61.5%	42.1%
	All three doses timely	2.2%	14.2%	27.7%	29.2%	31.1%	35.6%	23.8%
Median age in weeks at vaccination, IQR	Dose 1	17.3 (10.1–32.4)	11.3 (7.9–17.4)	8.6 (7.1–11.3)	8.6 (7.3–10.9)	8.6 (7.1–11.0)	8.4 (7.0–10.9)	9.1 (7.3– 13.0)
	Dose 2	22.1 (15.9–33.7)	18.7 (14.1–26.9)	14.7 (12.3–18.3)	14.6 (12.3–18.3)	14.3 (12.1–18.6)	14.1 (12.0–17.3)	15.3 (12.4– 19.7)
	Dose 3	24.1 (19.1–32.7)	25.5 (20.0–34.4)	20.9 (17.4–25.9)	21.0 (17.9–26.0)	21.0 (17.4–26.4)	20.4 (17.3–24.3)	21.3 (17.9– 26.9)

*Average calculated from all 20 clinic catchment basins, weighted by eligible population size. ^For one-dose coverage infants 6 weeks (42 days) or older; for two-dose coverage infants 10 weeks (70 days) or older; For 3 dose coverage infants 14 weeks (98 days) or older. ^&^These coverage estimates with the total number of respondents as the denominator (i.e. not just age-eligible infants) are shown for comparison with other estimate that do not adjust for age eligibility. †Infants whose first dose of 13-valent pneumococcal conjugate vaccine (PCV13) vaccination took place when they were 4 weeks to 2 months old (28 to 60 days old). ¶Infants whose second dose of PCV13 vaccination took place when they were 8 weeks to 4 months old (56 to 121 days old). §Infants whose third dose of PCV13 vaccination took place when they were 12 weeks to 6 months old (84 to 182 days old). IQR, interquartile range.

**Figure 2.**
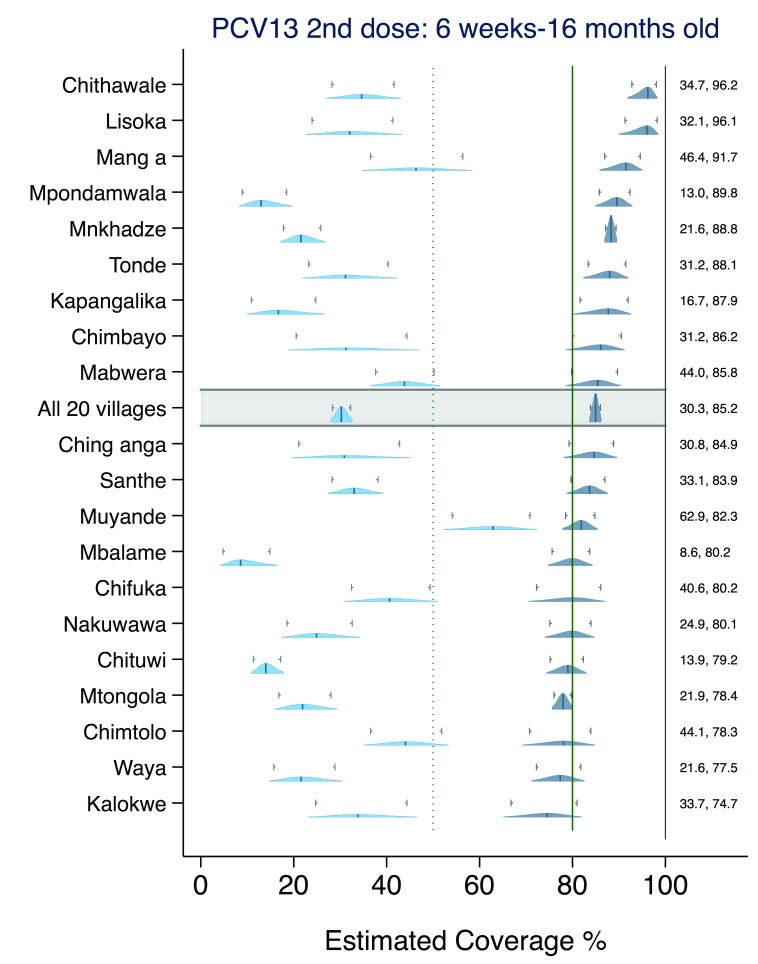
The two-dose coverage of 13-valent pneumococcal conjugate vaccine by lot and survey. Light blue, wave 1 (March 2012); dark blue, wave 6 (June 2014). Numbers on the right indicate the estimated coverage at waves 1 and 6. Tick marks indicate one-sided 95% confidence interval bounds. Green vertical target lines at 50% (dashed) and 80% (solid).

Figure 2 shows how the coverage of the first two doses of PCV13 changed in each basin from the first survey in March 2012 to the sixth and last survey in June 2014 in 6-week to 16-month-old babies. In March 2012, all but one (Muyande) of the 20 basins had a two-dose coverage below 50%, as indicated by the confidence intervals for the estimated coverage being below, or overlapping 50% coverage. By June 2014 all of the basins had coverage above 50% and eight of the 20 basins had achieved ≥80% two-dose coverage, as indicated by the lower confidence interval for the estimated proportion vaccinated being above 80% ( Figure 2, basins in order of June 2014 coverage). All basins combined were also estimated to have a two-dose coverage above 80% in June 2014 ( Figure 2).

**Figure 3.**
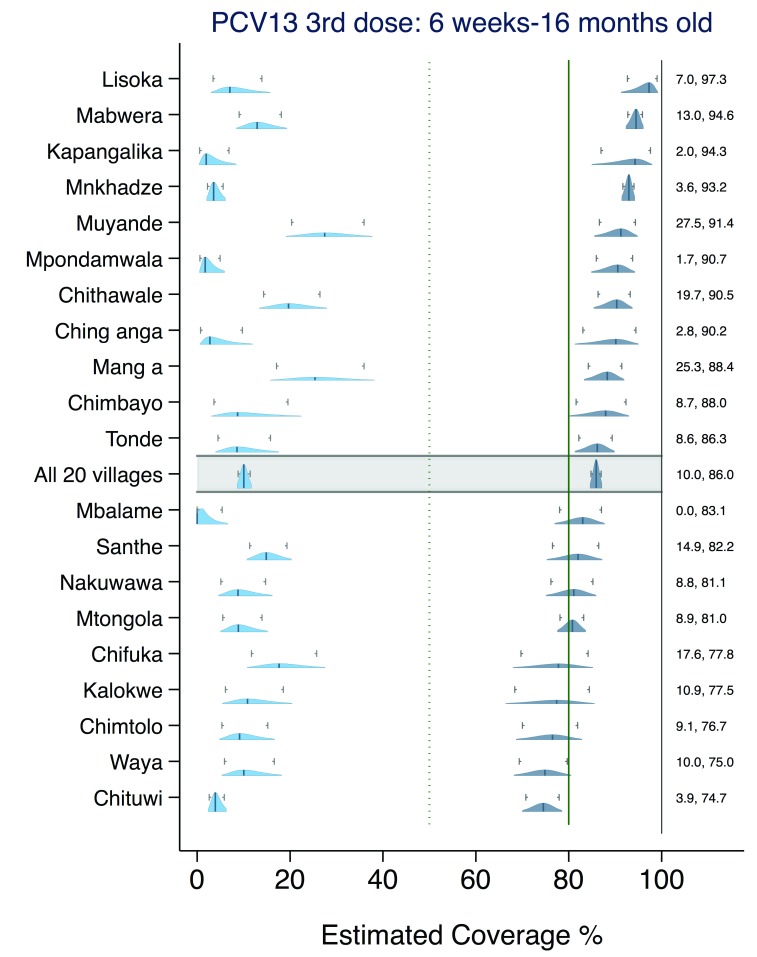
The three-dose coverage of 13-valent pneumococcal conjugate vaccine by lot and survey. Light blue, wave 1 (March 2012); dark blue, wave 6 (June 2014). Numbers on the right indicate the estimated coverage at waves 1 and 6. Tick marks indicate one-sided 95% confidence interval bounds. Green vertical target lines at 50% (dashed) and 80% (solid).

In March 2012 coverage of all three doses of PCV13 was more than 95% likely to be <40% in all 20 basins ( Figure 3). By June 2014 three-dose coverage was more than 95% likely to be ≥60% in all 20 basins, ≥70% in 16 basins, and met the ≥80% local target in 11 basins ( Figure 3).

The two-dose and three-dose PCV13 coverage was consistently lower in 6-week to 4-month-old infants than in 4–16-month-old infants ( Table 1 and Supplementary Figure 1– Supplementary Figure 4).

**Figure 4.**
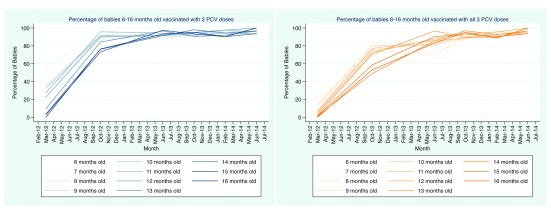
Trends in two-dose and three-dose coverage of 13-valent pneumococcal conjugate vaccine by age in months.

Figure 4 shows the trend for the overall two-dose and three-dose coverage by age, over-time. The March 2012 survey shows that the two-dose coverage was higher (≥50%) in infants aged 6 and 7 months old but lower (≤40%) in infants aged 8–16 months. The two-dose coverage increased to ≥90% in June 2014 for all infants aged 6–16 months. The three-dose coverage was lower (<20%) among all infants aged 6–16 months old in March 2012 but increased to >80% by October 2013 and >85% by June 2014. All raw data associated with this study are freely available from the UK Data Service ^17^.

## Discussion

Our results show that three-dose PCV13 vaccination coverage in Kabudula health area met the local district target of ≥80% ^8^ by the second year of the vaccination campaign, but only in older infants. By October 2012, the end of year 1 of the PCV vaccination program in the study area, the survey found that the overall three-dose coverage in age-eligible infants was at 64% (Infants <4 months old: 8%, Infants over 4 months old: 68%), far below the national target of ≥90% and the local district target of ≥80% ^8^. Our analysis shows that none of the basins had three-dose coverage of ≥80% after year 1 ( Supplementary Figure 5). The three-dose coverage in age-eligible infants increased to 80% (infants <4 months old, 9%; infants over 4 months old, 84%) by the end of year 2 (October 2013 survey), and 86% (infants <4 months old: 30%; infants over 4 months old: 90%) by June 2014, two-thirds through year 3 ( Table 1). In total, 14 of the 20 basins had >80% coverage in 4–16-month-olds in June 2014 ( Supplementary Figure 4).

We found timeliness of PCV13 vaccination to be low during the first year of the vaccination campaign, probably reflecting the initial catch-up campaign of vaccinating all infants under 1 year. By June 2013 we found that around half of infants were vaccinated within the standard schedule for each dose but only around one-quarter were receiving all three doses within the first 6 months of life as per schedule. This rose slightly by June 2014 to 49% for the first dose, 61% and 62% for the second and third doses and 36% for all three doses ( Table 1). This is lower than reported by a recent study in Karonga, which found ~80% of eligible infants were vaccinated with all three PCV13 doses by 26 weeks of age ^18^. Virtually all (99.8%) of the infants who were not vaccinated within the schedule were vaccinated late rather than early. Our results are comparable to the findings of Babirye *et al.* in Uganda, which used the same definitions of timeliness for the three doses of polio and pentavalent vaccination but found greater timeliness of each dose ranging from 71% for polio 1 to 78% for polio 2 and pentavalent 2 ^19^. In 2004 Malawi was found to have 52% coverage of DPT3 (diphtheria-pertussis-tetanus vaccination, third dose) by 6 months of age ^14^.

The three-doses-within-the-first-6-months schedule is recommended in settings such as Malawi, where severe vaccine-preventable disease is common in younger infants ^20^. Existing vaccine schedules at 6, 10 and 14 weeks, such as those for polio and pentavalent vaccine also make it logistically easier ^20^. Delayed vaccination extends individual susceptibility to illness and reduces herd immunity ^21^. However, whether an initial two-dose plus booster (2+1), initial three-dose (3+0) or a three-dose plus booster (3+1) schedule is better at preventing clinical pneumonia remains unresolved ^22^. A recent trial in South Africa, which has similar pneumonia epidemiology to Malawi, found a 2+1 PCV schedule of 6, 14, 38 weeks to significantly reduce IPD ^4^.

There are some limitations to this study. Firstly, the sample size for the infants aged 6 weeks to 4 months was less than half of the intended sample of 600 infants (30 per basin) for all the surveys conducted. This was due to there being insufficient infants of this age group in each basin at the time of survey. However, given the low numbers who were vaccinated and the high probability of low coverage thresholds not being met given the estimated confidence intervals, we can be fairly sure that the coverage was low in this age group. Secondly, the self-weighted analysis here implicitly assumes that every eligible respondent had the same chance of being selected, but this is not so in this case, as the interviewers always started at the centre of the village. Furthermore, for confidence intervals to be meaningful, the sample should be a probability sample and the analysis should incorporate appropriate sampling weights. This simple self-weighted analysis of a somewhat biased sample may not meet the strict requirements for confidence intervals. However, the six surveys used the same method of data collection each time, so the biases over time are likely to be constant. The coverage results also improved so dramatically over time that our broad-stroke conclusions are likely to be robust. The villages are small and a large proportion of the eligible children were sampled in each village so the improvements in coverage and the persistent shortfalls in timeliness described here are likely to be real, although subject to bias. Thirdly, due to a large proportion of eligible children being surveyed in some basins, some of the same children in the older 4–16-months age group may have been sampled in more than one survey, introducing some correlation to the repeated cross-sectional samples, meaning our assessment of trends in vaccine coverage may be less representative of the overall trend in the population. This is likely to be a minor issue given that the surveys were 4–8 months apart. Fourthly, the studies were only conducted in one of the six health areas of Lilongwe district; therefore the results may not be generalizable, unless compared with other studies carried out during the same period in other parts of the country. Kabudula is however, fairly representative of rural Lilongwe district and Malawi in general, being found to have vaccination rates around the average for the whole country in 2007, although variation in local vaccine stocks and logistics is an important consideration ^23^. Fifthly, although 96% of infants had vaccination data photographed from health passports, meaning the potential of recall bias to affect our results is low, there were a few anecdotal reports of vaccination records being filled before vaccinations were administered. Vaccine stock-outs at clinics or health facilities mean it was possible that a health passport could indicate vaccination when the infant was not vaccinated. From discussions with health facility and immunization staff, we were assured that this was a relatively rare occurrence. Mothers also verbally verified their infants were vaccinated in almost all cases where vaccination was indicated on the infant’s health passport. Finally, tracking coverage in each basin can be difficult—the same basins did not always have lower coverage ( Supplementary Figure 5), likely due to changing community and health-system dynamics, as well as due to artefacts of the relatively small sample sizes in each basin.

Before the advent of PCV13, it was estimated that PCV13 would protect against 63% of all circulating invasive pneumococci and 78% of those found in children under 5 years in Malawi ^24^. We previously published that at 76% three-dose PCV13 coverage, compared to 0% three-dose coverage, and among children <59 months of age, cases of pneumonia associated with low oxygen levels (i.e., hypoxemia) decreased by nearly 50% and hospital pneumonia fatalities declined by more than one-third in central Malawi ^9^. Furthermore, the proportion of pneumonia cases with clinical danger signs associated with high risk of mortality fell by nearly two-thirds in Malawi after the introduction of PCV13 ^9^.

## Conclusion

Meeting and maintaining the 90% PCV13 coverage target in all areas of Malawi in the coming months and years, and improving the timeliness of vaccination in young infants, has great potential to reduce the burden of PCV13-preventable disease. Monitoring the progress towards this goal via household surveys of PCV13 coverage is important.

## Data availability

All aggregate data is reported in the manuscript and supplementary files. Individual anonymized data from all six waves of the survey and the Stata software code used to produce all tables and figures is freely available via the UK Data Service: https://doi.org/10.5255/UKDA-SN-853265 ^17^. This UK Data Service deposit also contains the data collection tool.

Data are available under the terms of the Creative Commons Attribution 4.0 International license (CC-BY 4.0).

## Supplementary material

**Supplementary Figure 1. The two-dose coverage of PCV13 in 6-week to 4-month-old babies by lot and survey.**

.

**Supplementary Figure 2. The two-dose coverage of PCV13 in 4–16 month old infants by lot and survey.**

.

**Supplementary Figure 3. The three-dose coverage of PCV13 in 6 week to 4 month old babies by lot and survey.**

.

**Supplementary Figure 4. The three-dose coverage of PCV13 in 4–16 month old infants by lot and survey.**

.

**Supplementary Figure 5. PCV13 coverage in each village clinic basin for each survey wave (W), for each age cohort (1, <4 months old; 2, 4–16 months old) and for both cohorts combined.**
