# Supplementary figures and images for: An assessment of PCV13 vaccine coverage using a repeated cross-sectional household survey in Malawi
Source: Gates Open Res. 2018 Aug 2;2:37. [Version 1] doi: 10.12688/gatesopenres.12837.1 (PMC6266718; doi:10.12688/gatesopenres.12837.1)

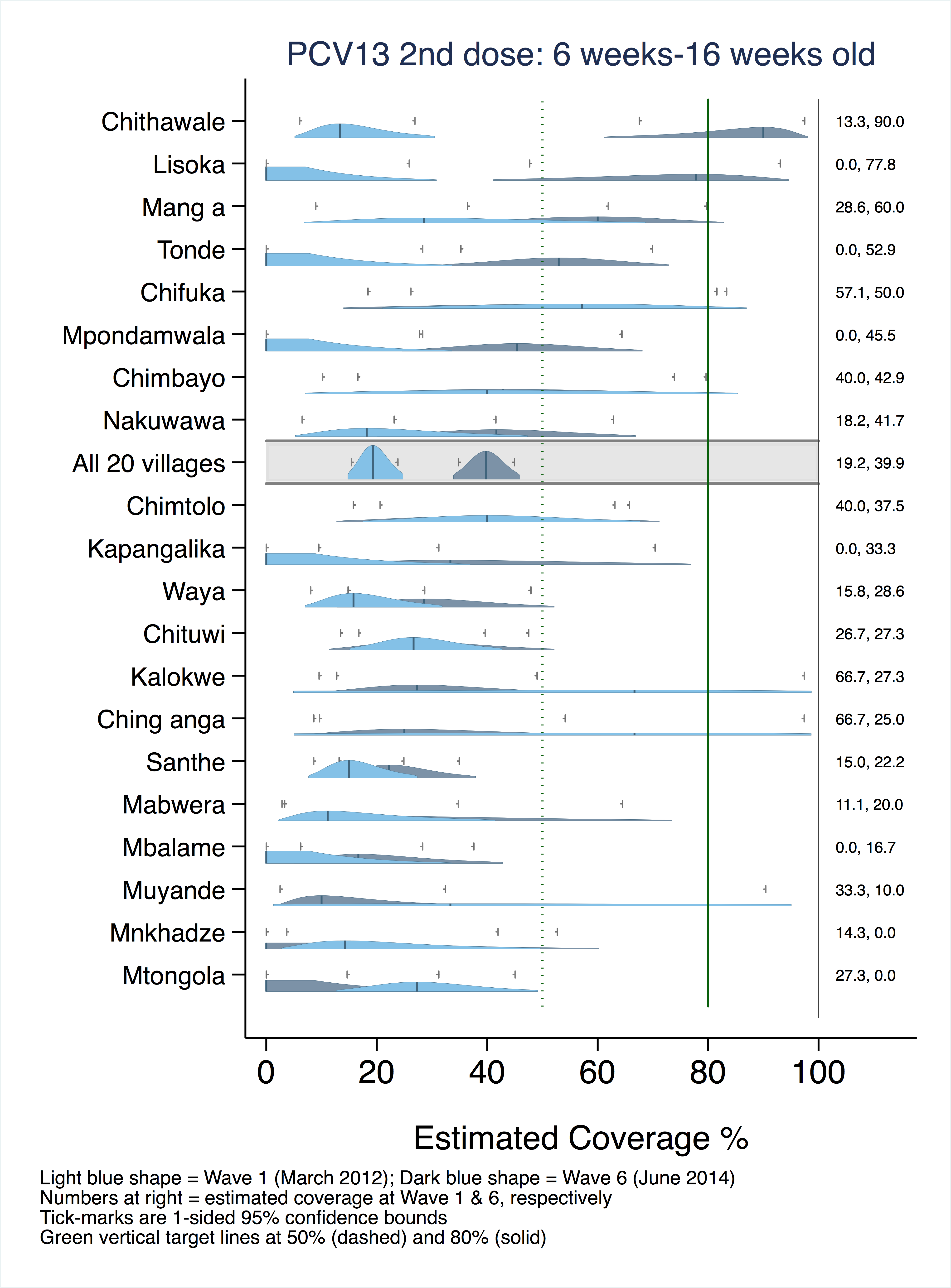

Supplement: Supplementary file 1 [file gatesopenres-2-13914-s0000.tgz › b61ef285-991e-4d36-986c-cd8ade4331ba.png]

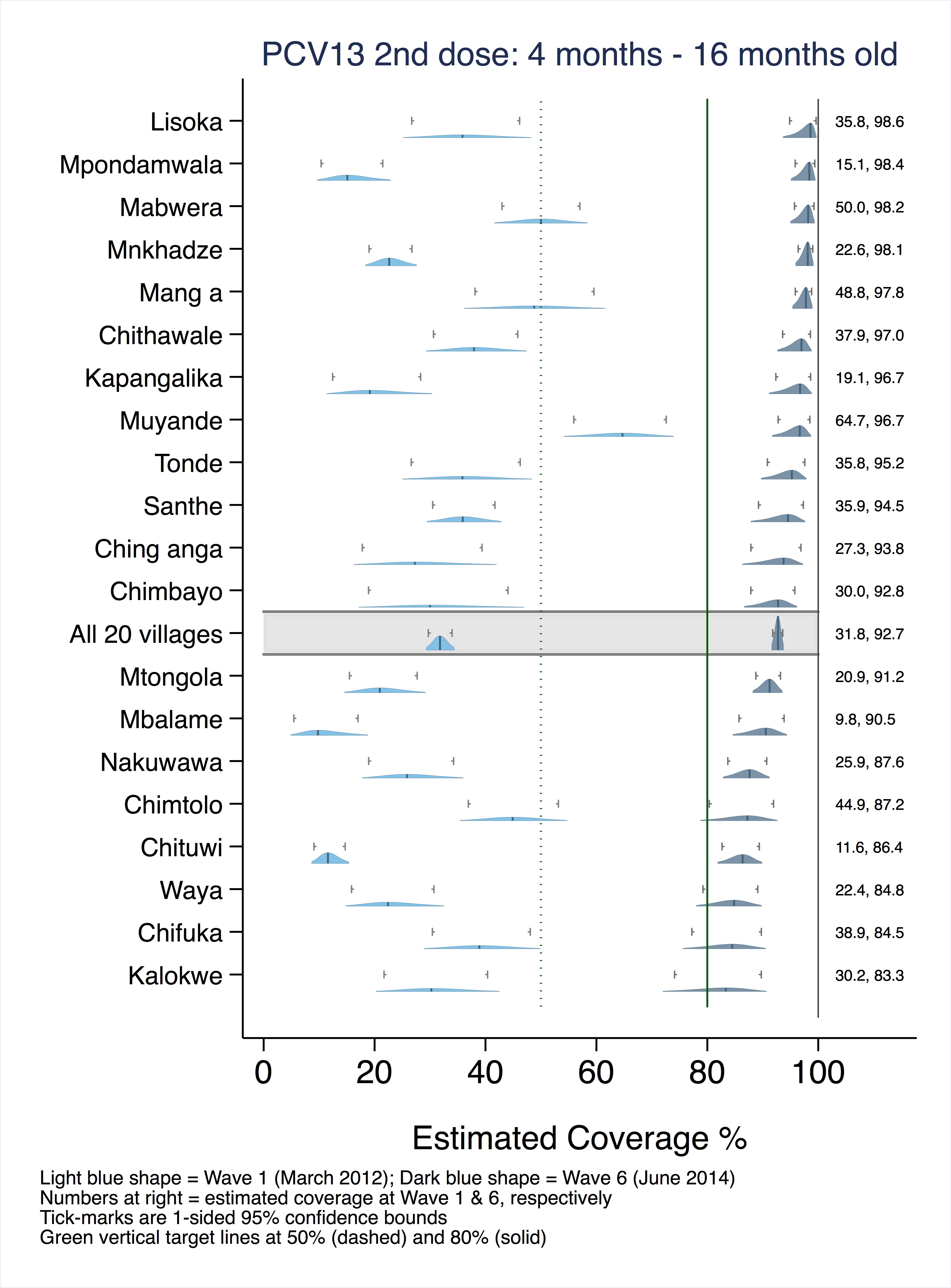

Supplement: Supplementary file 2 [file gatesopenres-2-13914-s0001.tgz › b060c0b5-c11f-4568-b238-29977522e45d.png]

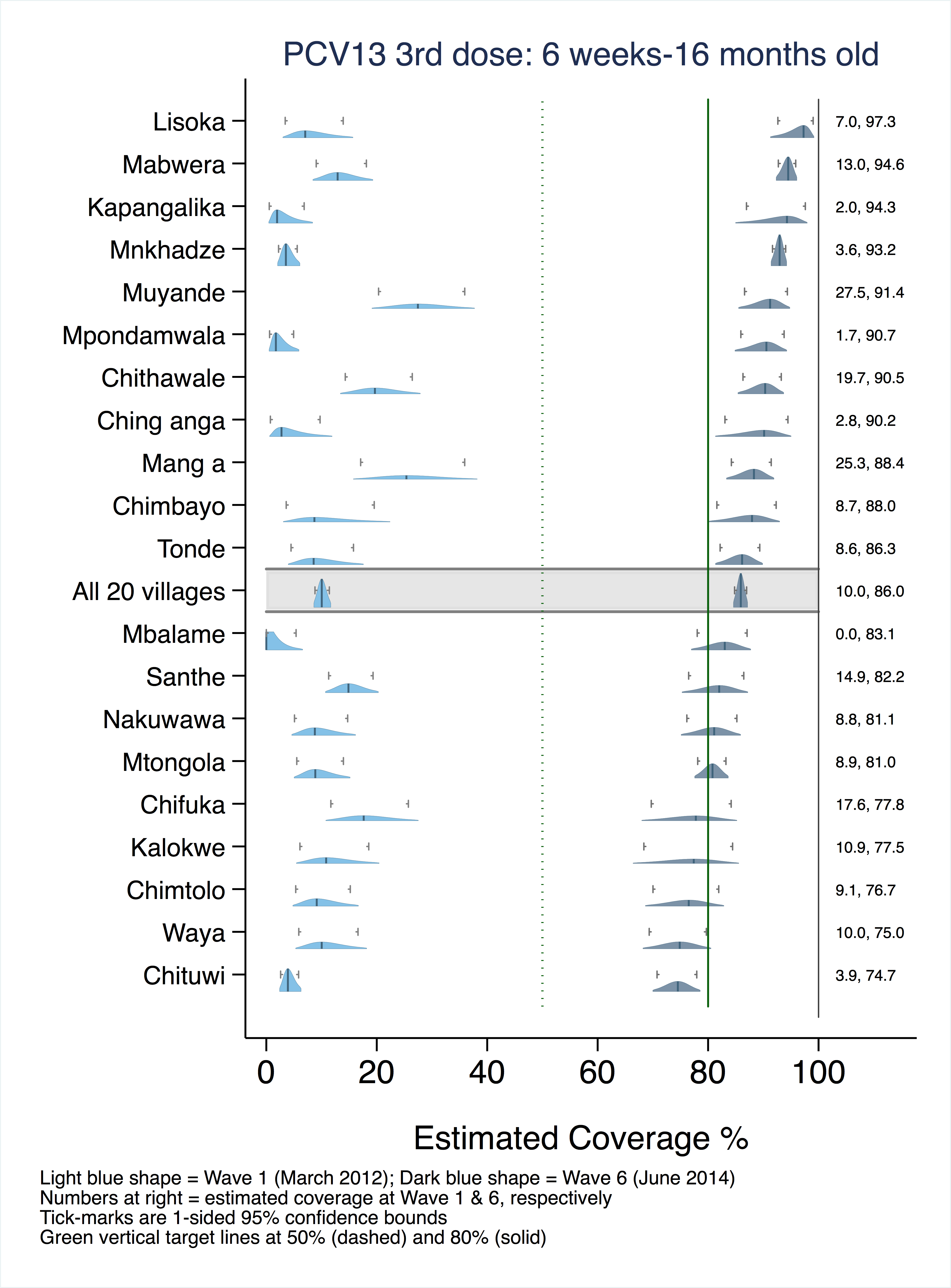

Supplement: Supplementary file 3 [file gatesopenres-2-13914-s0002.tgz › 2bb027bd-6fb3-4d8b-b365-aaf681efabcd.png]

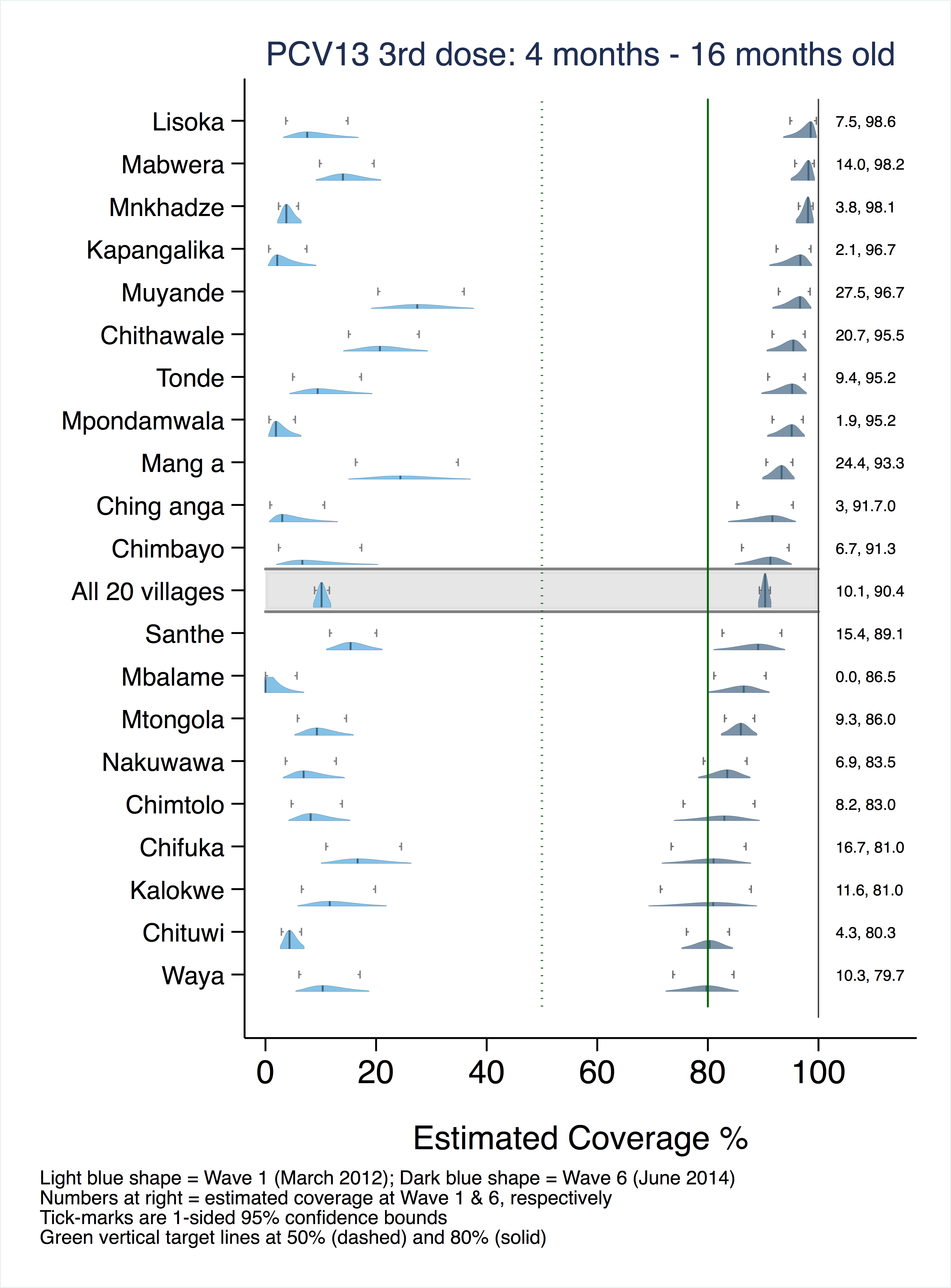

Supplement: Supplementary file 4 [file gatesopenres-2-13914-s0003.tgz › f5e2b6f6-a0d6-4935-9e21-9d277e4fb37c.png]
