# Supplementary material for: An assessment of PCV13 vaccine coverage using a repeated cross-sectional household survey in Malawi
Source: Gates Open Res. 2018 Aug 2;2:37. [Version 1] doi: 10.12688/gatesopenres.12837.1 (PMC6266718; doi:10.12688/gatesopenres.12837.1)
Supplement: Supplementary file 5 [file gatesopenres-2-13914-s0004.tgz › 72d2f8e9-3972-40cf-9033-58569572ea38.pdf]

Supplementary Figure 5: a) Coverage in Chifuka village clinic basin in each survey wave (W) for each age cohort (1=<4 months old; 2=4-16 months old) and both cohorts combined

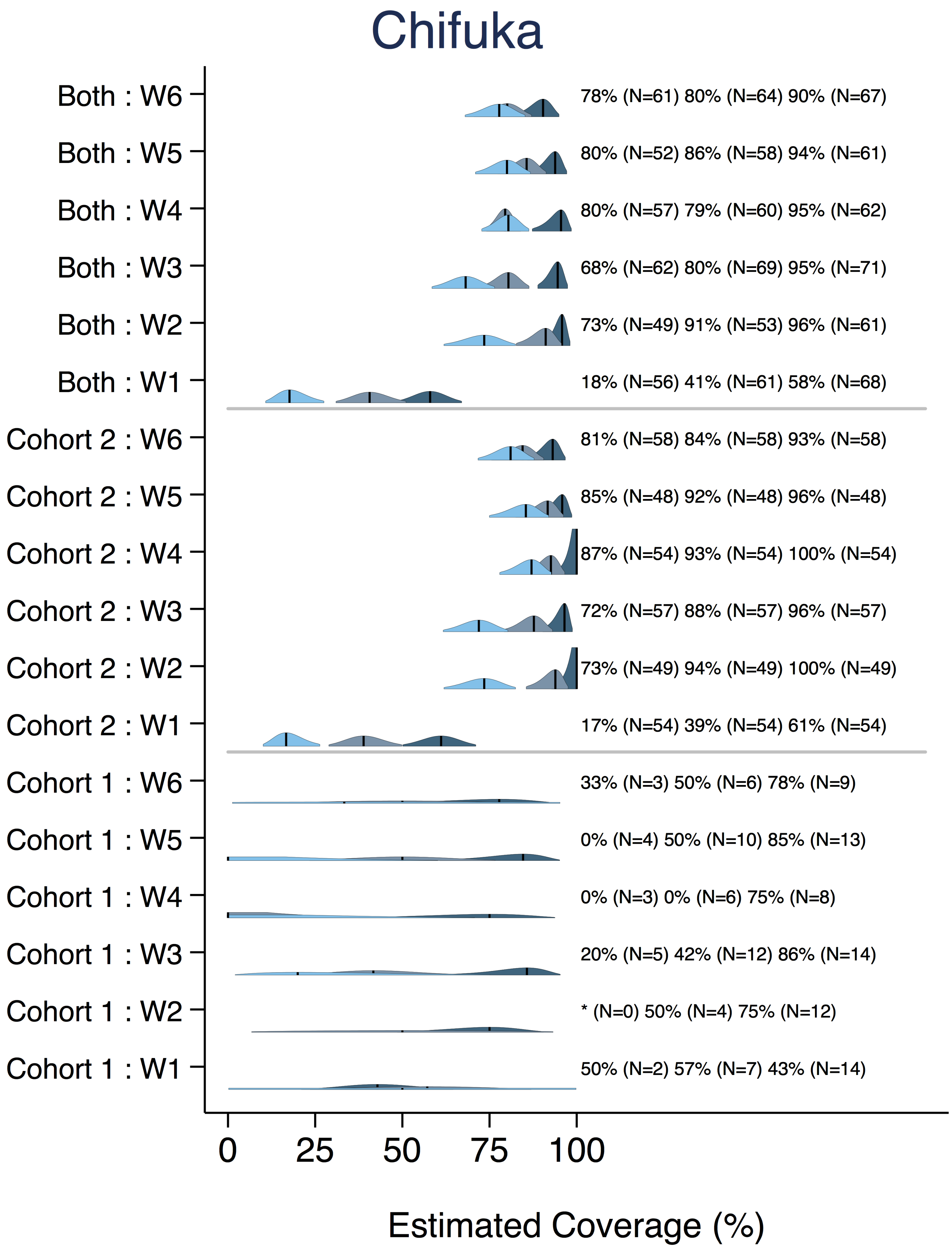

Text at right: Estimated coverge and sample size for doses 3, 2 and 1, respectively

b) Coverage in Chimbayo village clinic basin in each survey wave (W) for each age cohort (1=<4 months old; 2=4-16 months old) and both cohorts combined

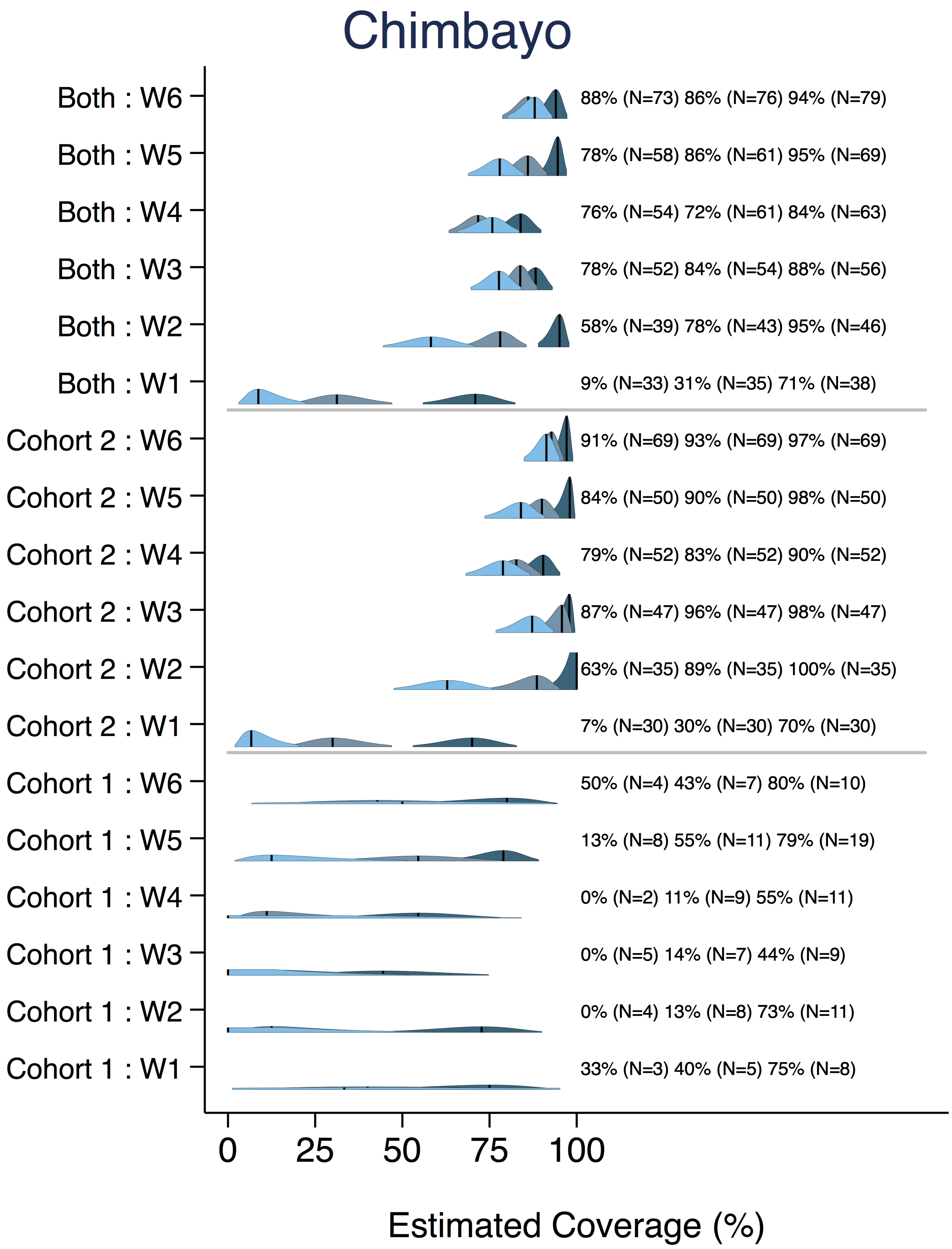

Text at right: Estimated coverge and sample size for doses 3, 2 and 1, respectively

c) Coverage in Chimtolo village clinic basin in each survey wave (W) for each age cohort (1=<4 months old; 2=4-16 months old) and both cohorts combined

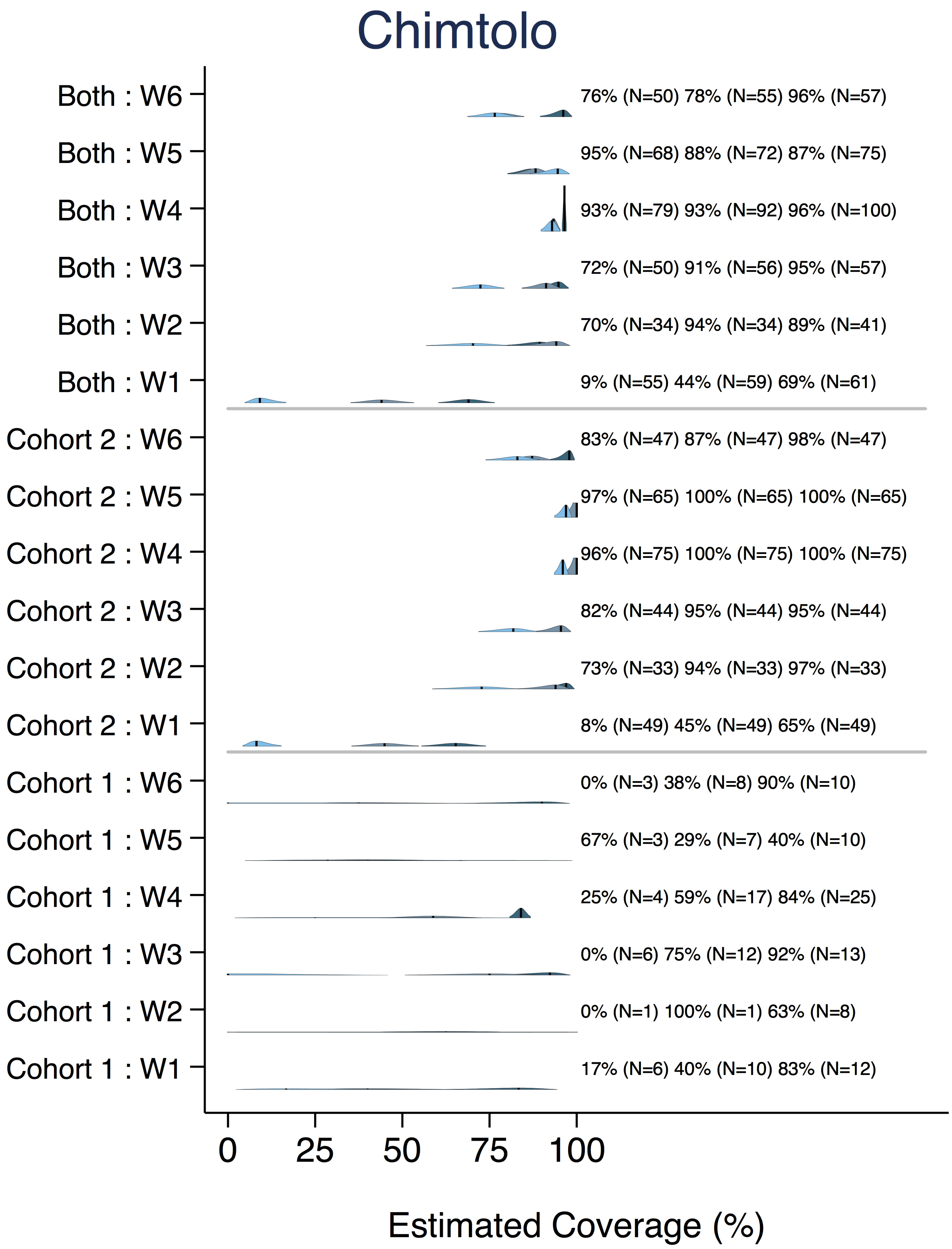

Text at right: Estimated coverge and sample size for doses 3, 2 and 1, respectively

d) Coverage in Ching'anga village clinic basin in each survey wave (W) for each age cohort (1=<4 months old; 2=4-16 months old) and both cohorts combined

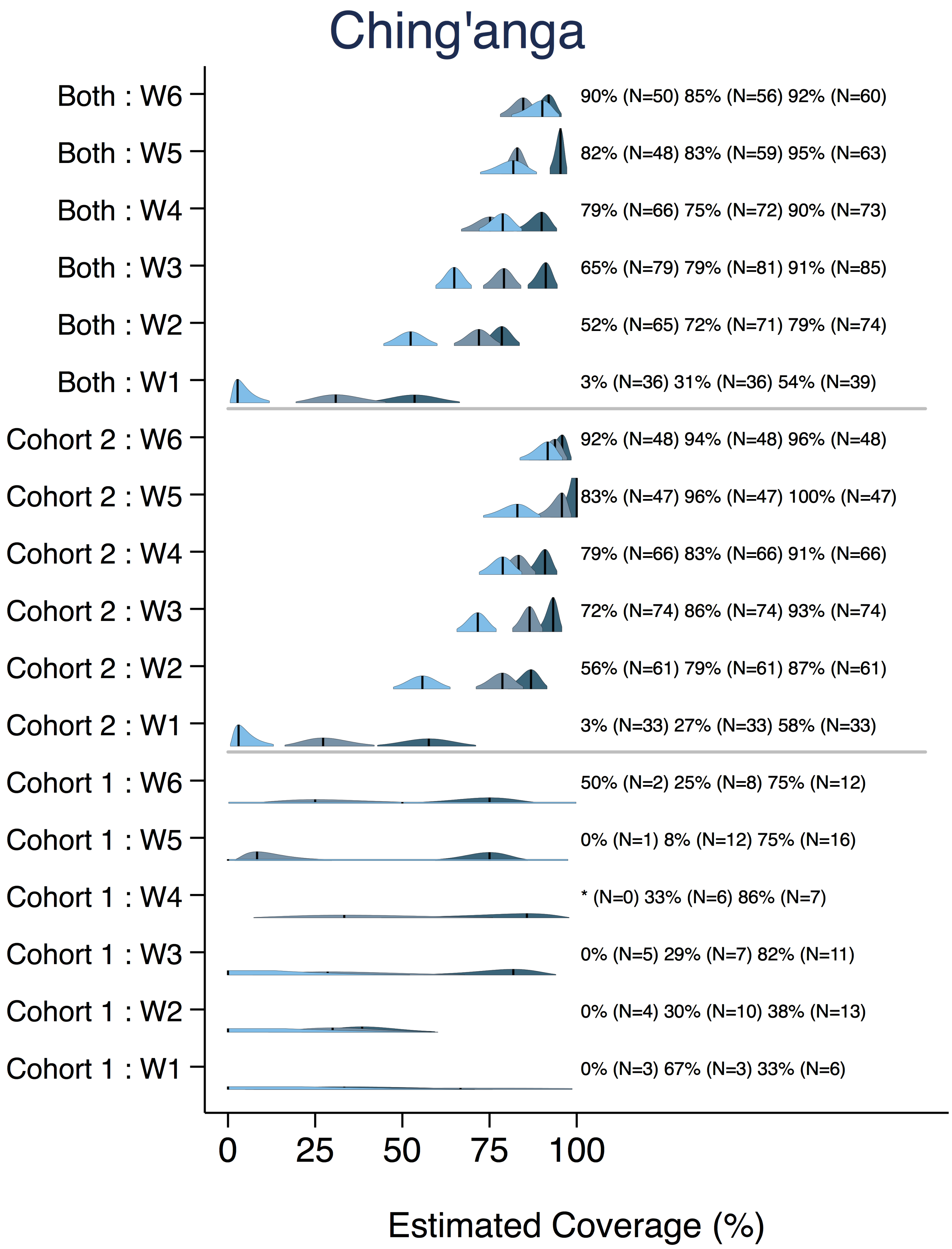

Text at right: Estimated coverge and sample size for doses 3, 2 and 1, respectively

e) Coverage in Chithawale village clinic basin in each survey wave (W) for each age cohort (1=<4 months old; 2=4-16 months old) and both cohorts combined

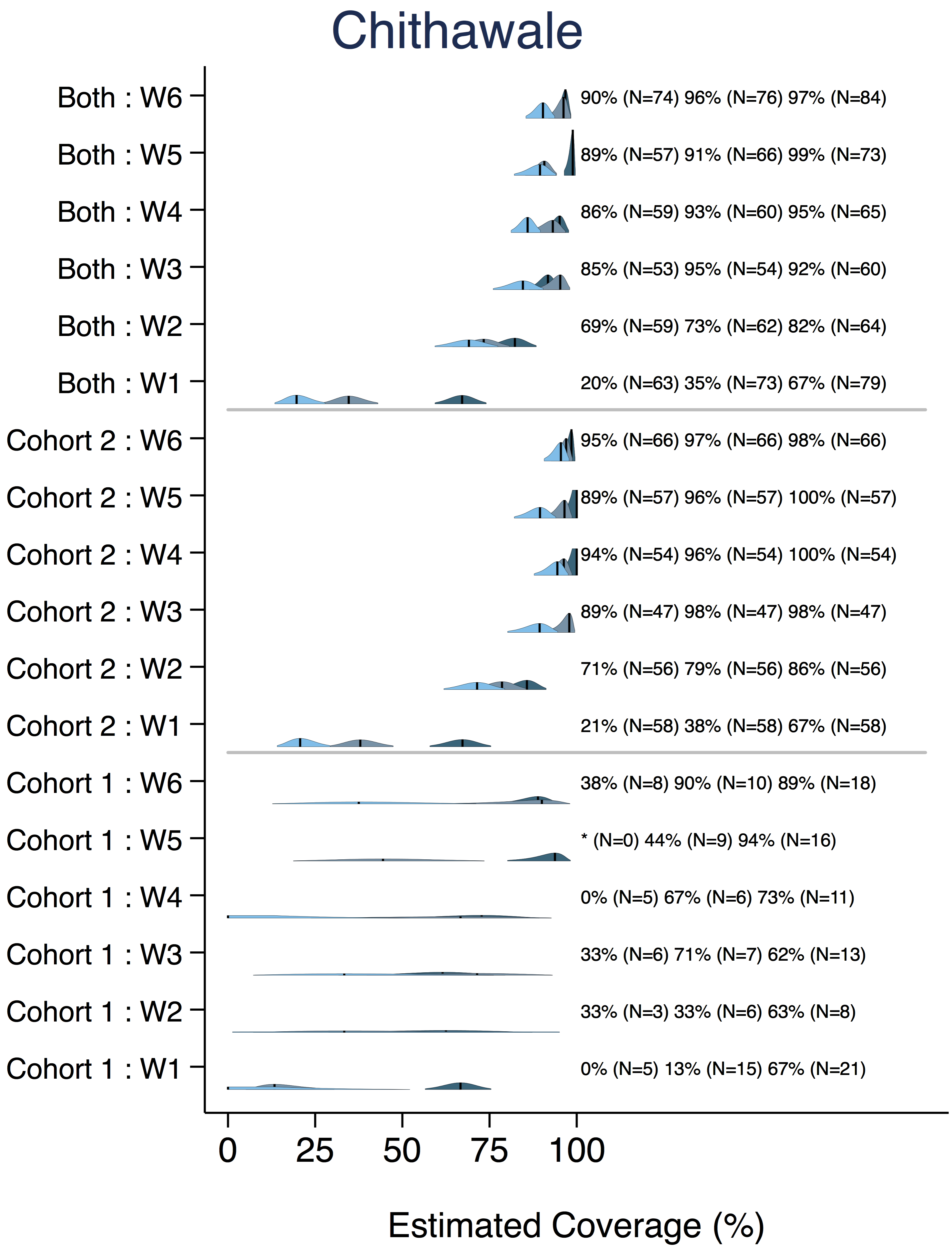

Text at right: Estimated coverge and sample size for doses 3, 2 and 1, respectively

f) Coverage in Chituwi village clinic basin in each survey wave (W) for each age cohort (1=<4 months old; 2=4-16 months old) and both cohorts combined

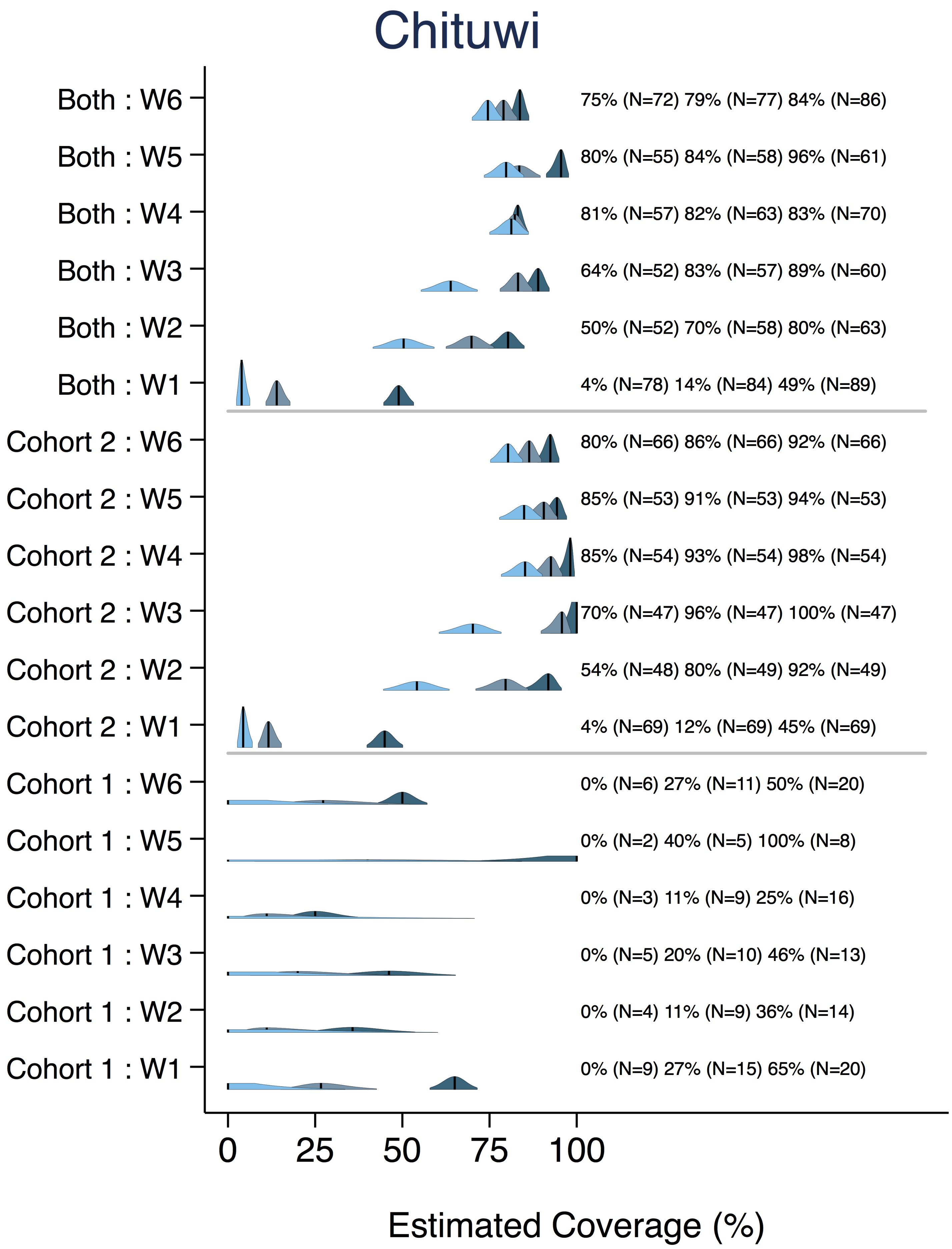

Text at right: Estimated coverge and sample size for doses 3, 2 and 1, respectively

g) Coverage in Kalokwe village clinic basin in each survey wave (W) for each age cohort (1=<4 months old; 2=4-16 months old) and both cohorts combined

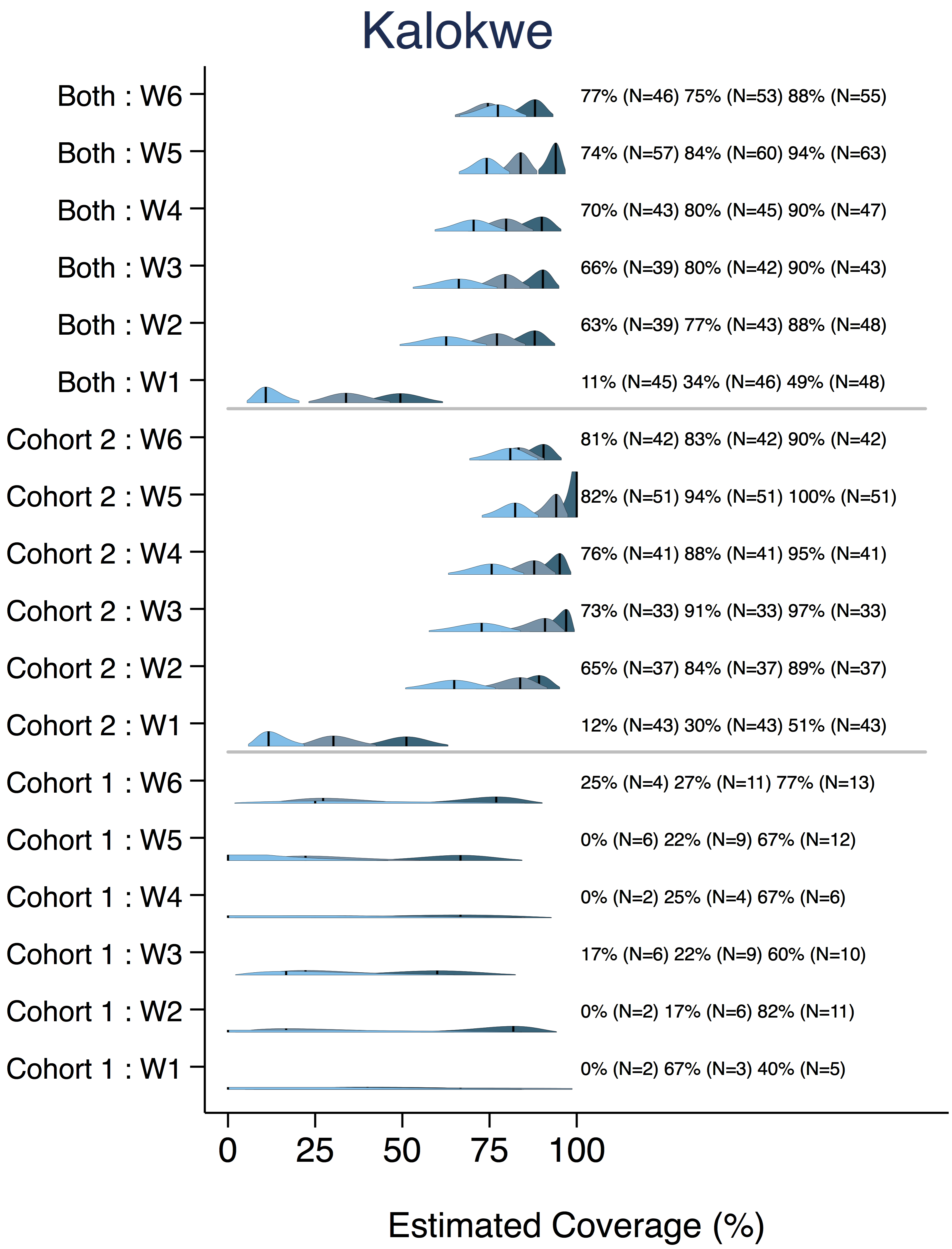

Text at right: Estimated coverge and sample size for doses 3, 2 and 1, respectively

h) Coverage in Kapangalika village clinic basin in each survey wave (W) for each age cohort (1=<4 months old; 2=4-16 months old) and both cohorts combined

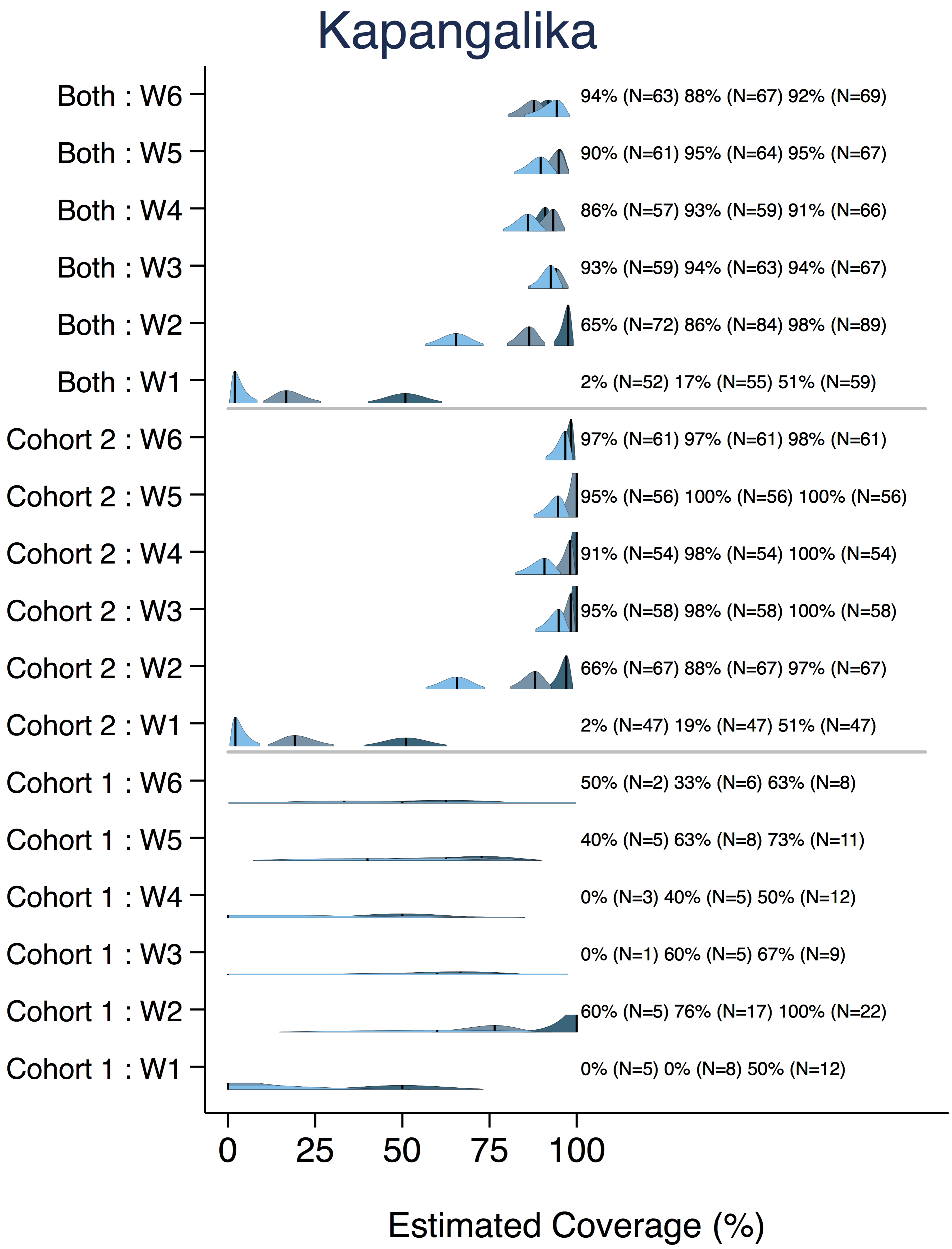

Text at right: Estimated coverge and sample size for doses 3, 2 and 1, respectively

i) Coverage in Lisoka village clinic basin in each survey wave (W) for each age cohort (1=<4 months old; 2=4-16 months old) and both cohorts combined

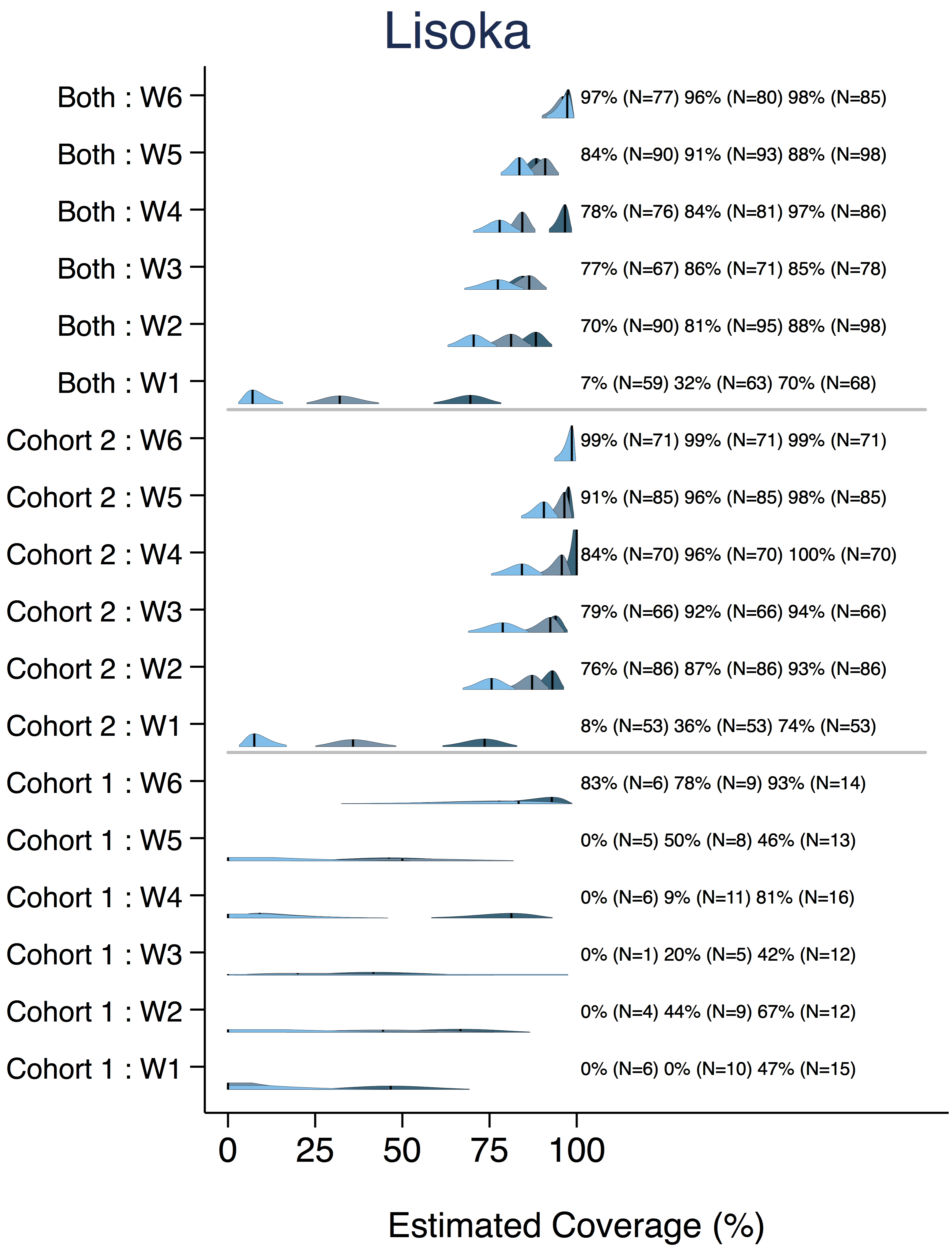

Text at right: Estimated coverge and sample size for doses 3, 2 and 1, respectively

j) Coverage in Mabwera village clinic basin in each survey wave (W) for each age cohort (1=<4 months old; 2=4-16 months old) and both cohorts combined

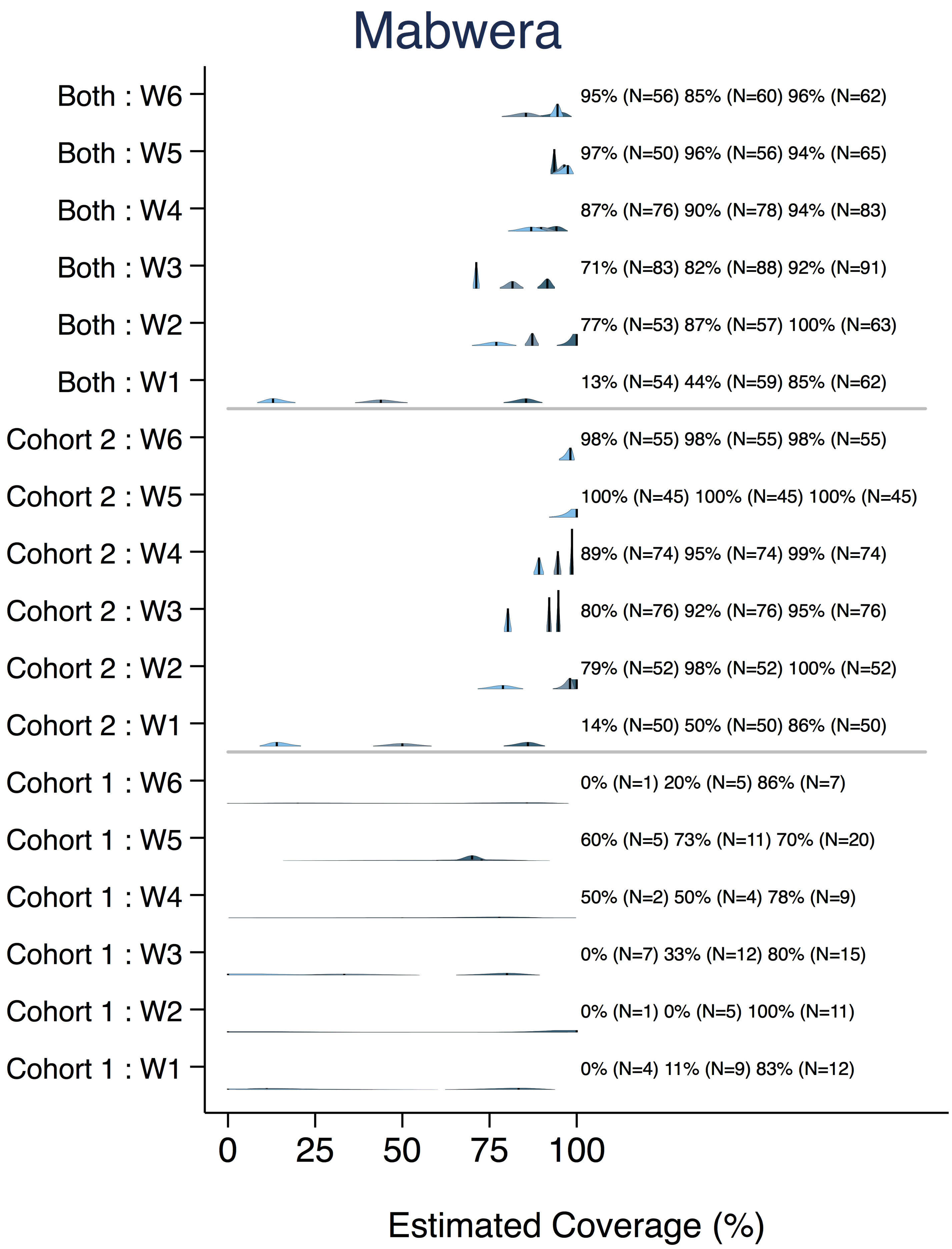

Text at right: Estimated coverge and sample size for doses 3, 2 and 1, respectively

k) Coverage in Mang'a village clinic basin in each survey wave (W) for each age cohort (1=<4 months old; 2=4-16 months old) and both cohorts combined

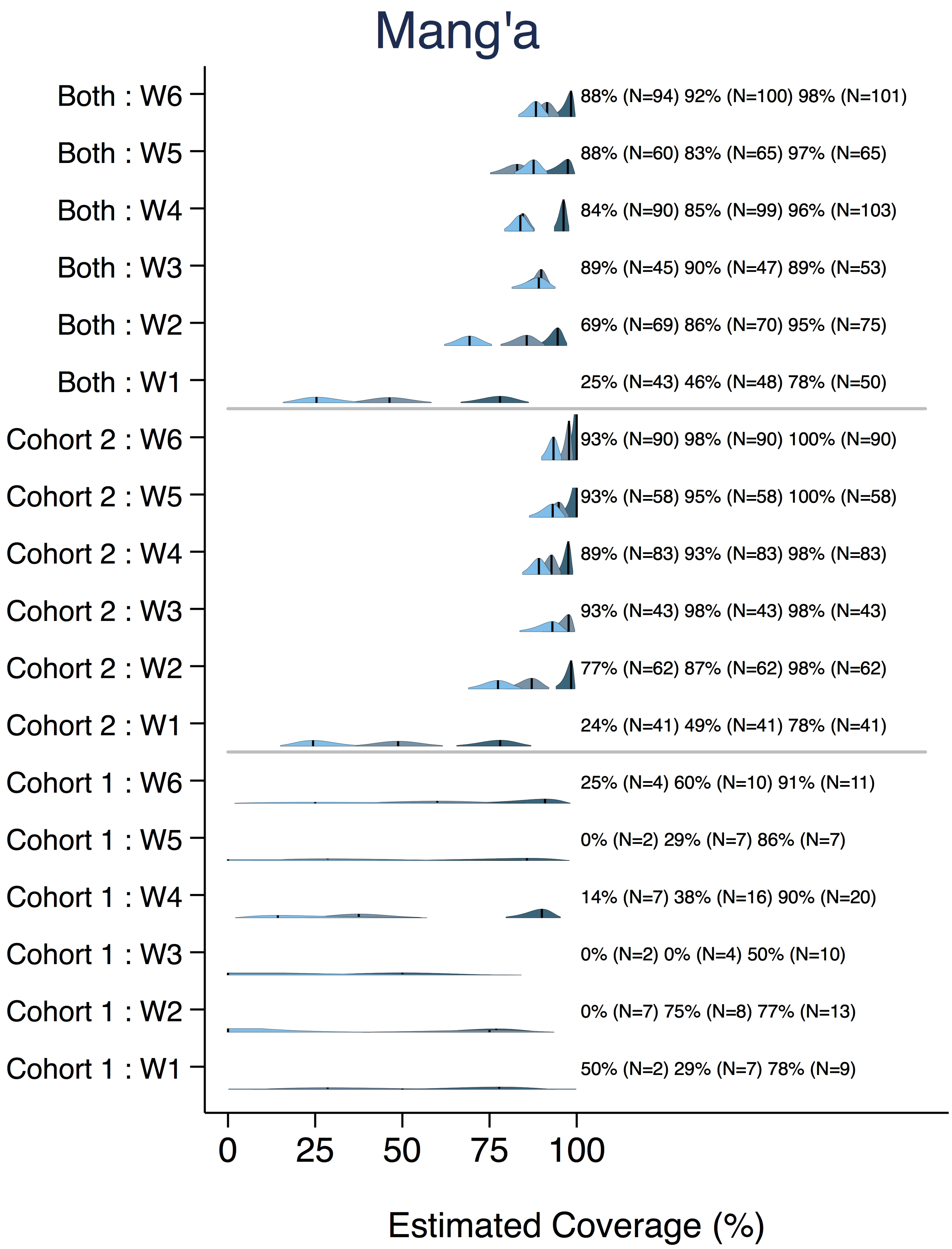

Text at right: Estimated coverge and sample size for doses 3, 2 and 1, respectively

I) Coverage in Mbalame village clinic basin in each survey wave (W) for each age cohort (1=<4 months old; 2=4-16 months old) and both cohorts combined

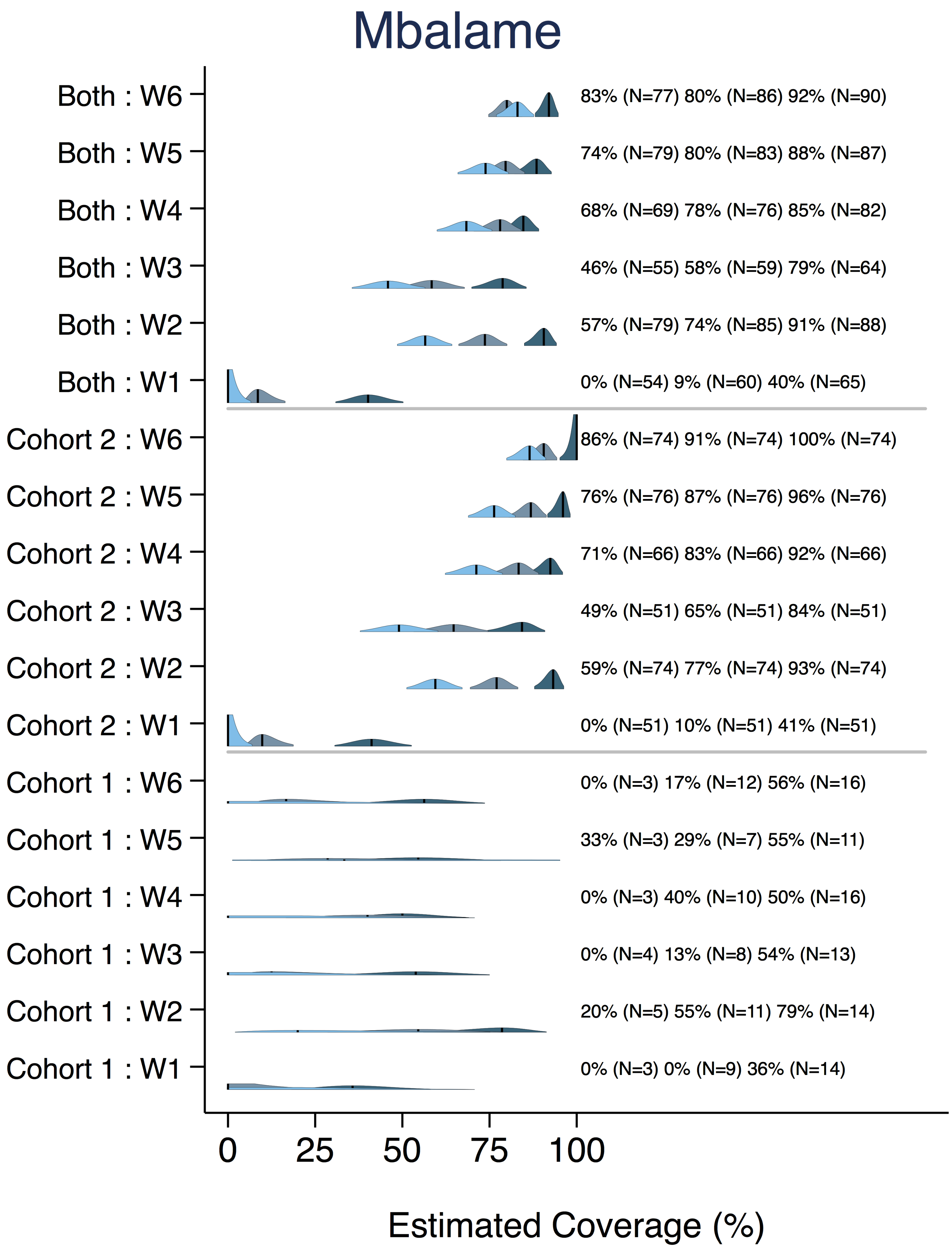

Text at right: Estimated coverge and sample size for doses 3, 2 and 1, respectively

m) Coverage in Mnkhadze village clinic basin in each survey wave (W) for each age cohort (1=<4 months old; 2=4-16 months old) and both cohorts combined

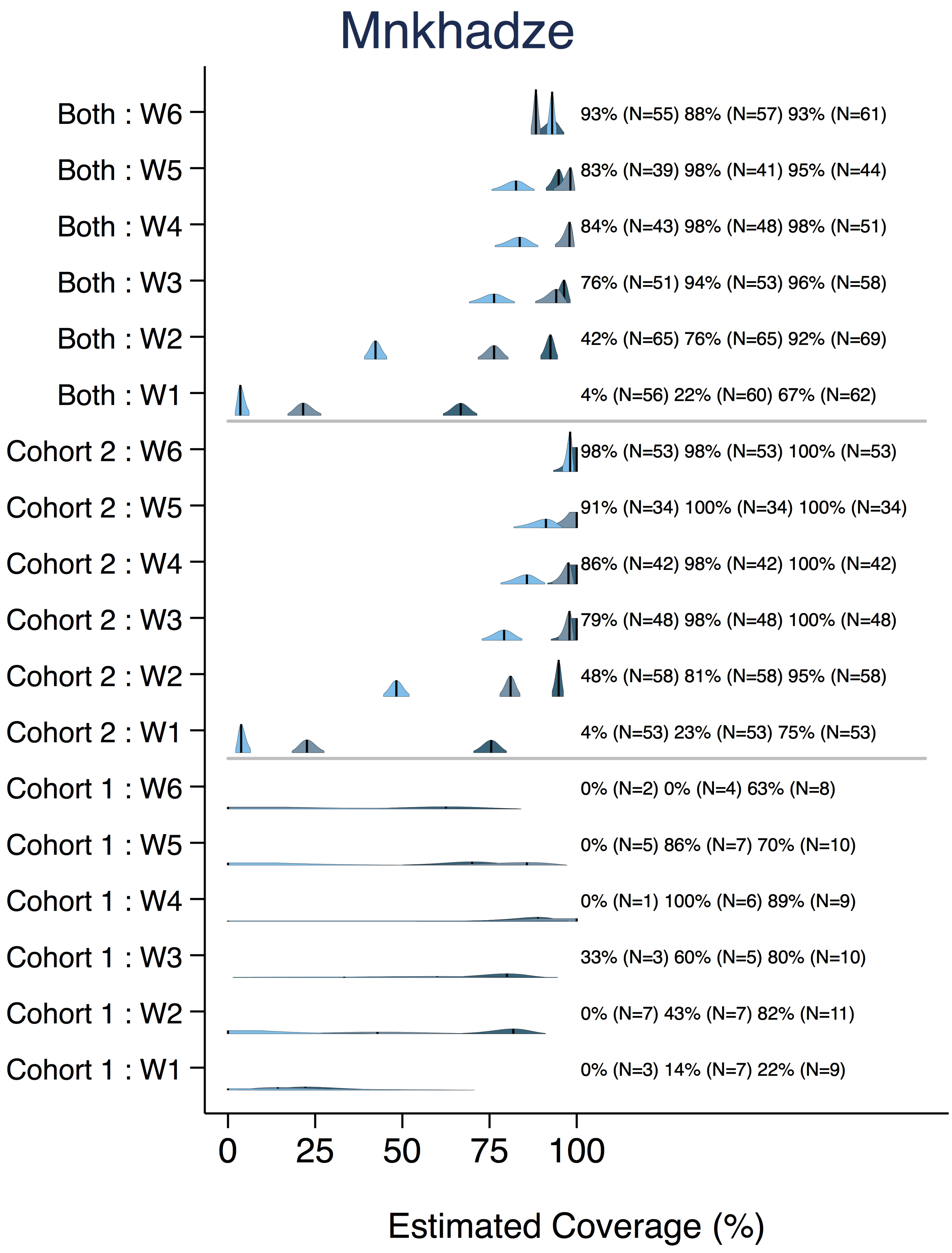

Text at right: Estimated coverge and sample size for doses 3, 2 and 1, respectively

n) Coverage in Mpondamwala village clinic basin in each survey wave (W) for each age cohort (1=<4 months old; 2=4-16 months old) and both cohorts combined

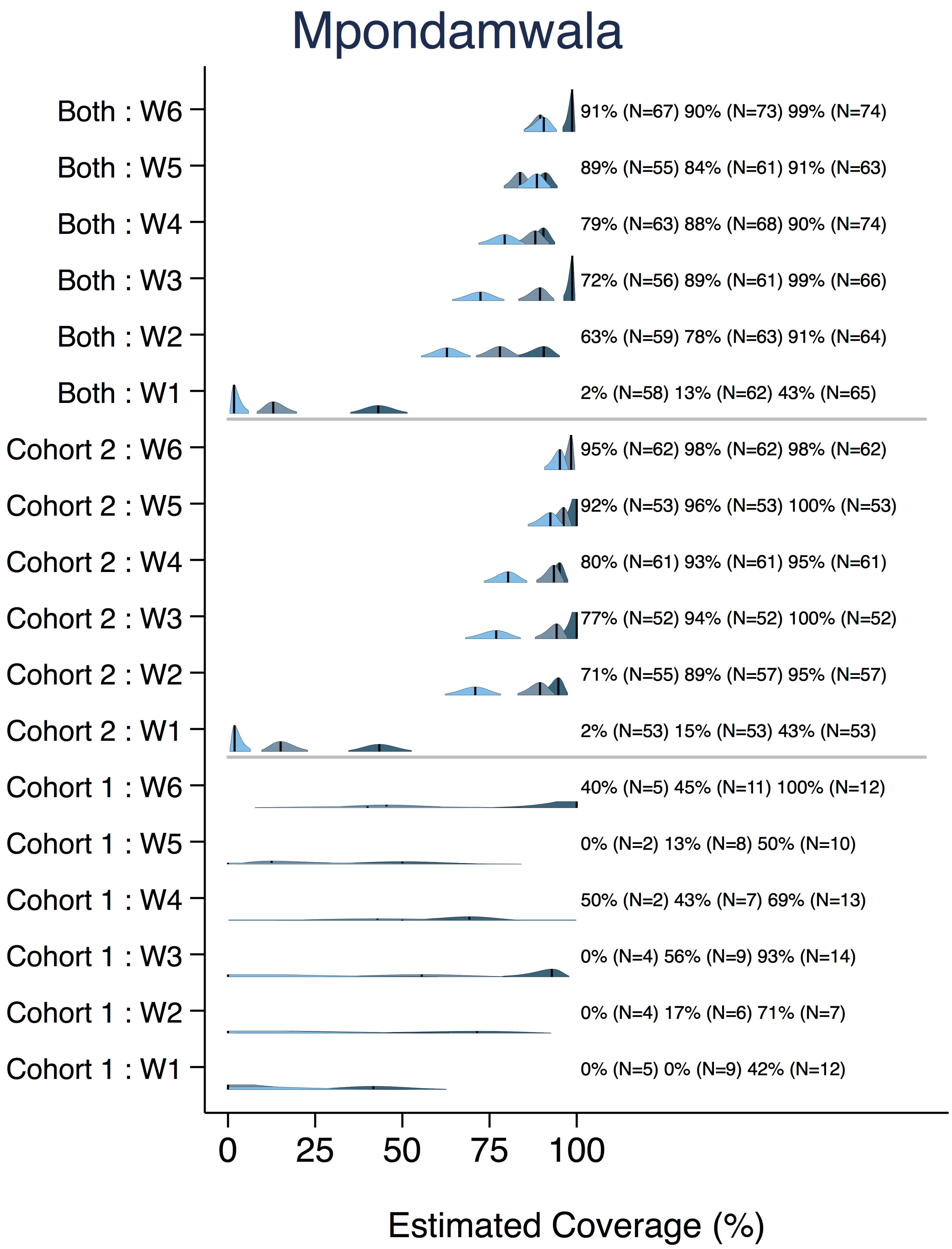

Text at right: Estimated coverge and sample size for doses 3, 2 and 1, respectively

o) Coverage in Mtongola village clinic basin in each survey wave (W) for each age cohort (1=<4 months old; 2=4-16 months old) and both cohorts combined

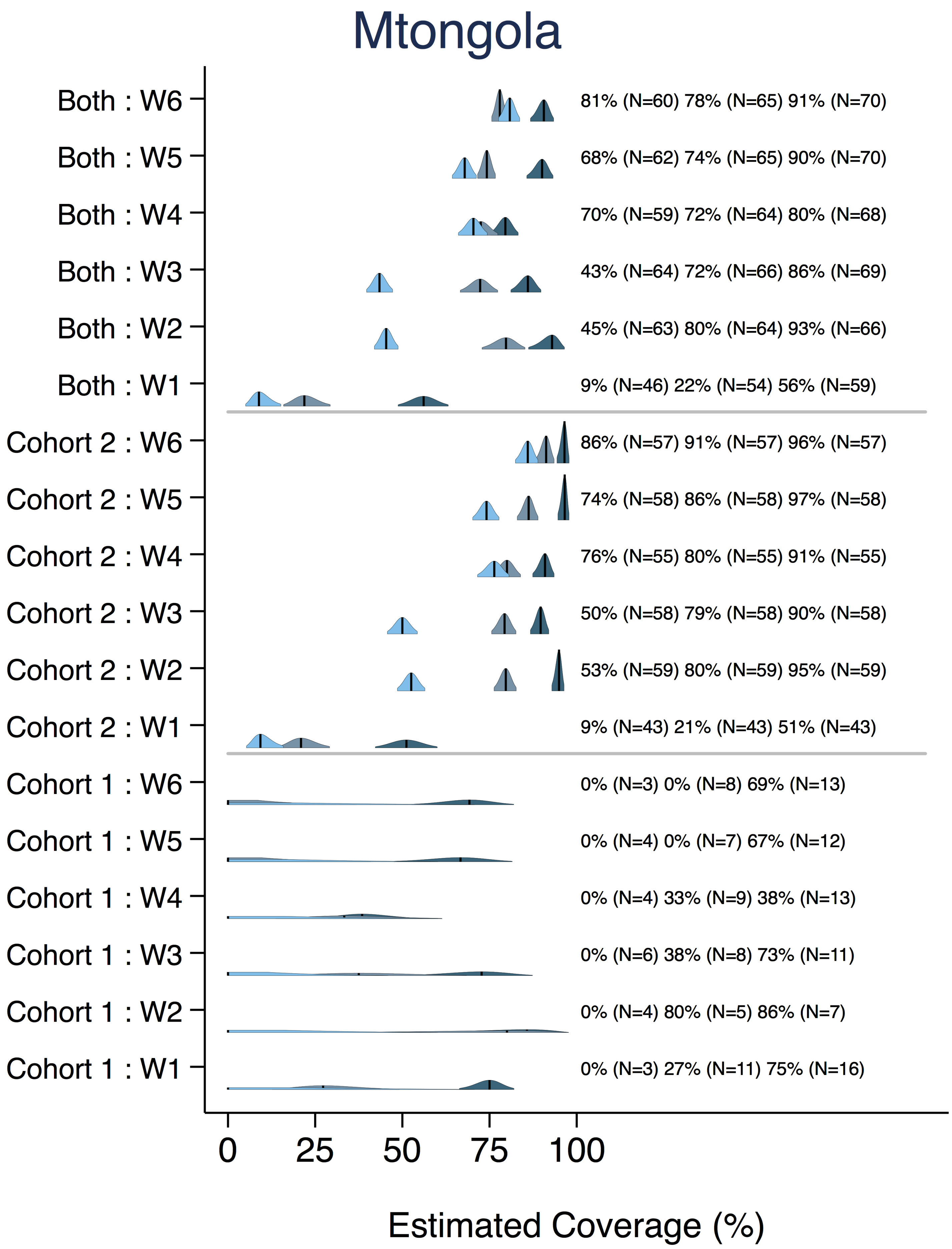

Text at right: Estimated coverge and sample size for doses 3, 2 and 1, respectively

p) Coverage in Muyande village clinic basin in each survey wave (W) for each age cohort (1=<4 months old; 2=4-16 months old) and both cohorts combined

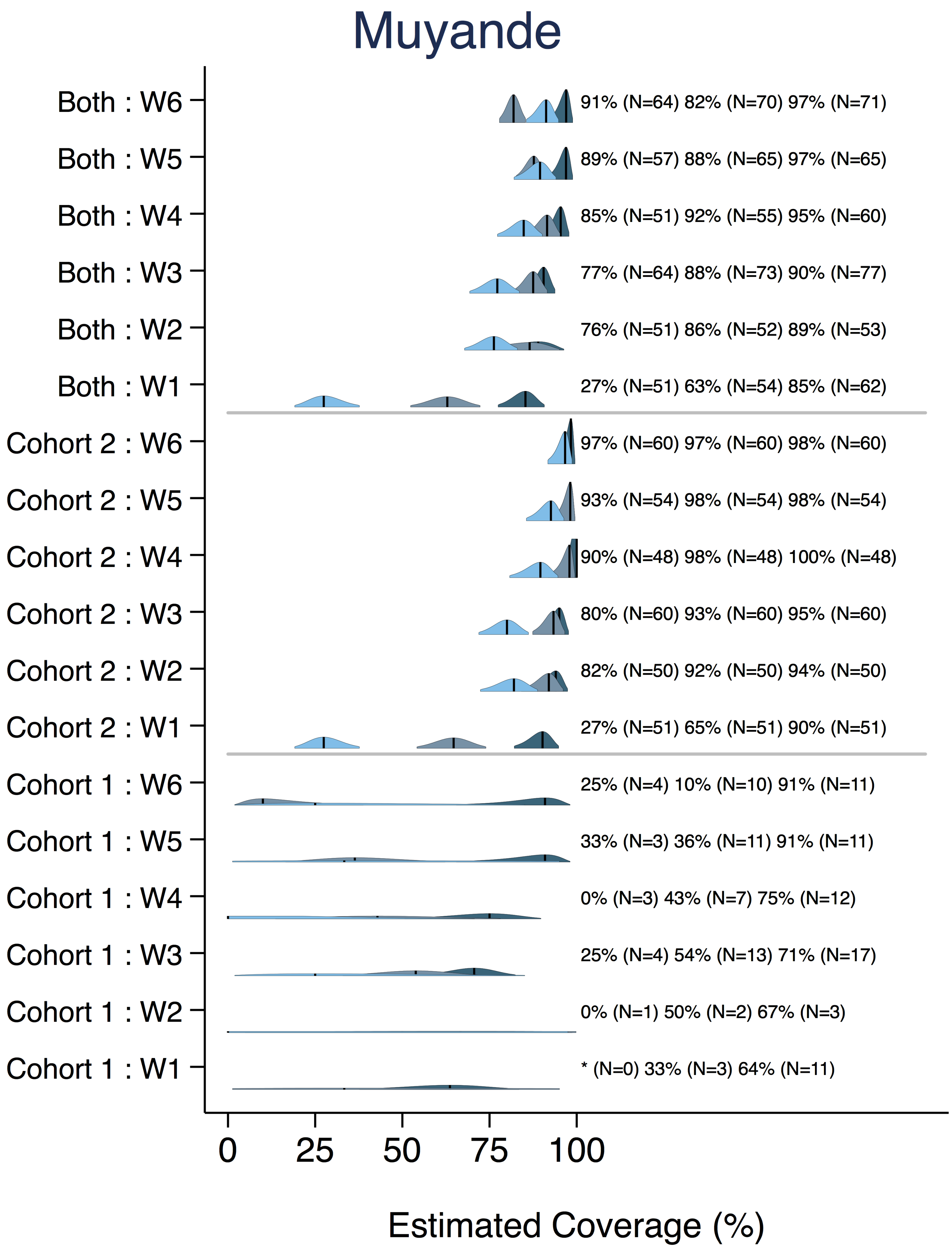

Text at right: Estimated coverge and sample size for doses 3, 2 and 1, respectively

q) Coverage in Nakuwawa village clinic basin in each survey wave (W) for each age cohort (1=<4 months old; 2=4-16 months old) and both cohorts combined

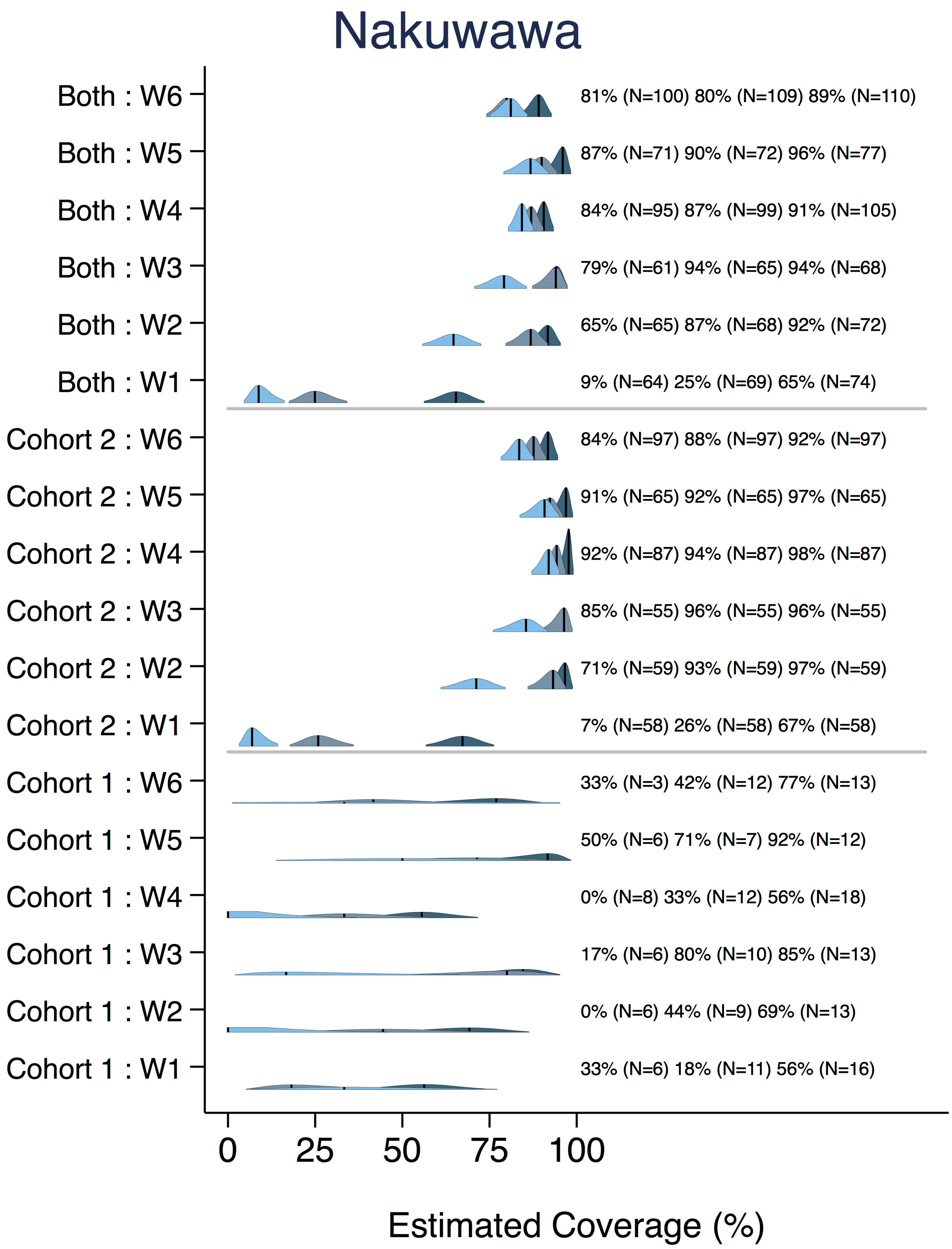

Text at right: Estimated coverge and sample size for doses 3, 2 and 1, respectively

r) Coverage in Santhe village clinic basin in each survey wave (W) for each age cohort (1=<4 months old; 2=4-16 months old) and both cohorts combined

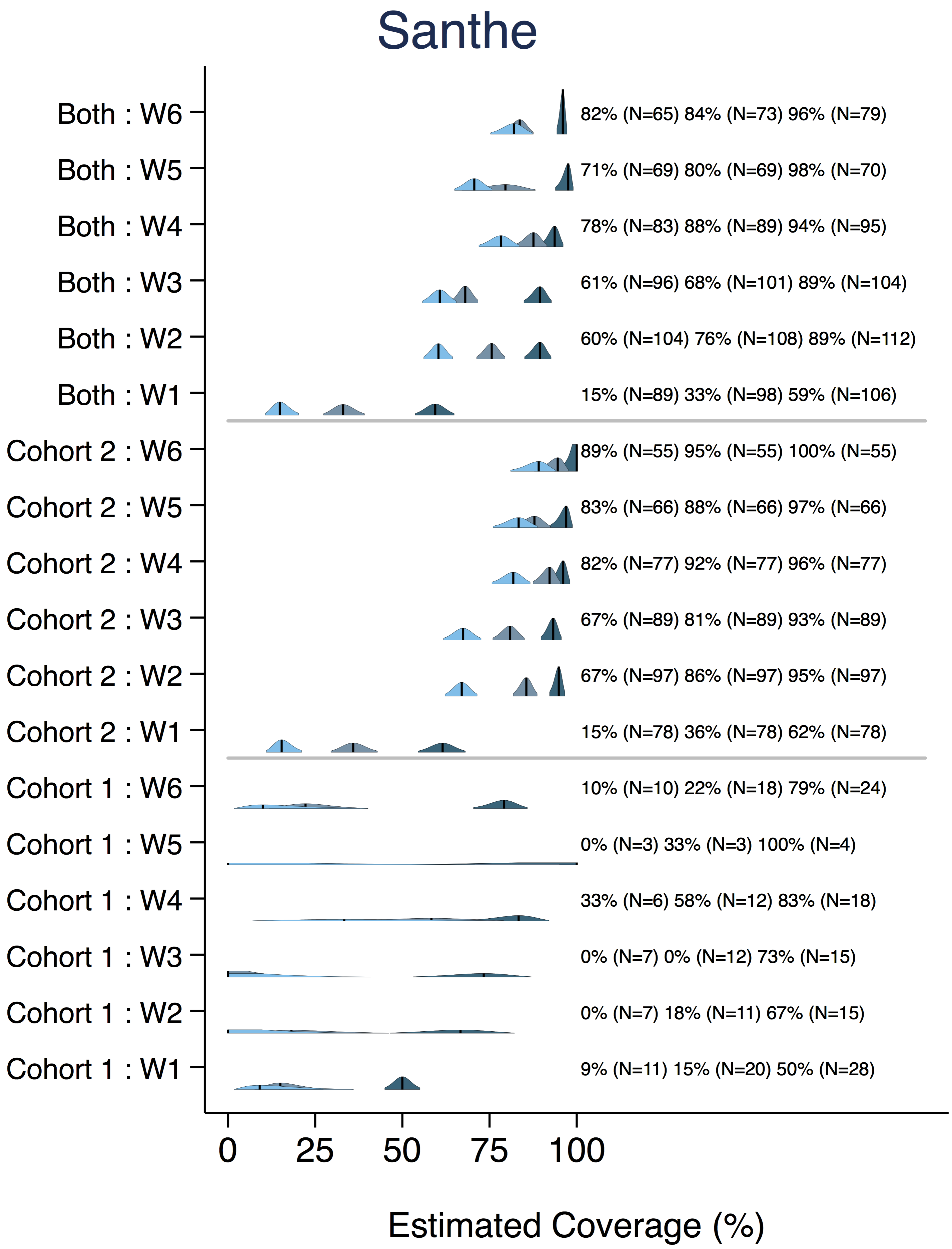

Text at right: Estimated coverge and sample size for doses 3, 2 and 1, respectively

s) Coverage in Tonde village clinic basin in each survey wave (W) for each age cohort (1=<4 months old; 2=4-16 months old) and both cohorts combined

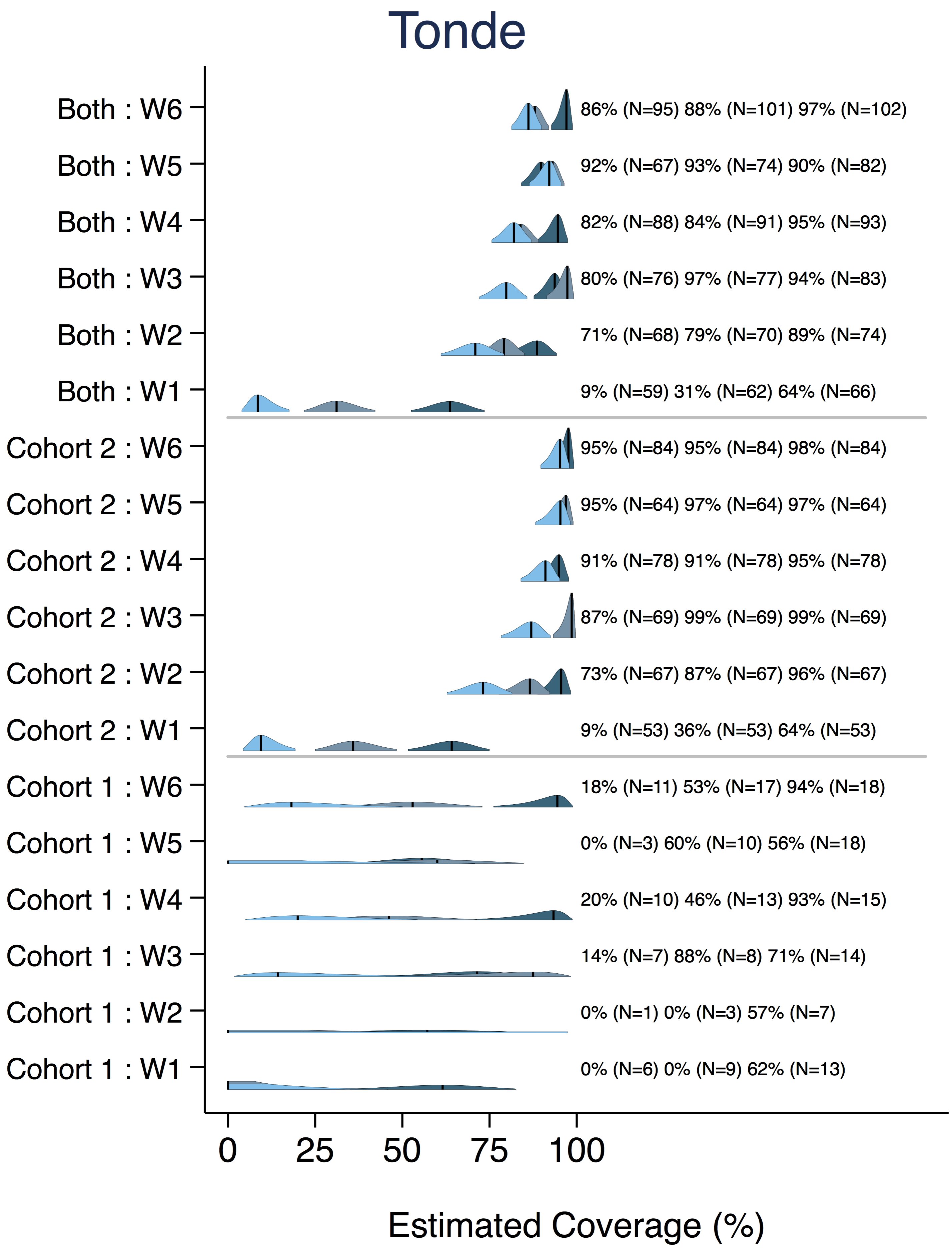

Text at right: Estimated coverge and sample size for doses 3, 2 and 1, respectively

t) Coverage in Waya village clinic basin in each survey wave (W) for each age cohort (1=<4 months old; 2=4-16 months old) and both cohorts combined

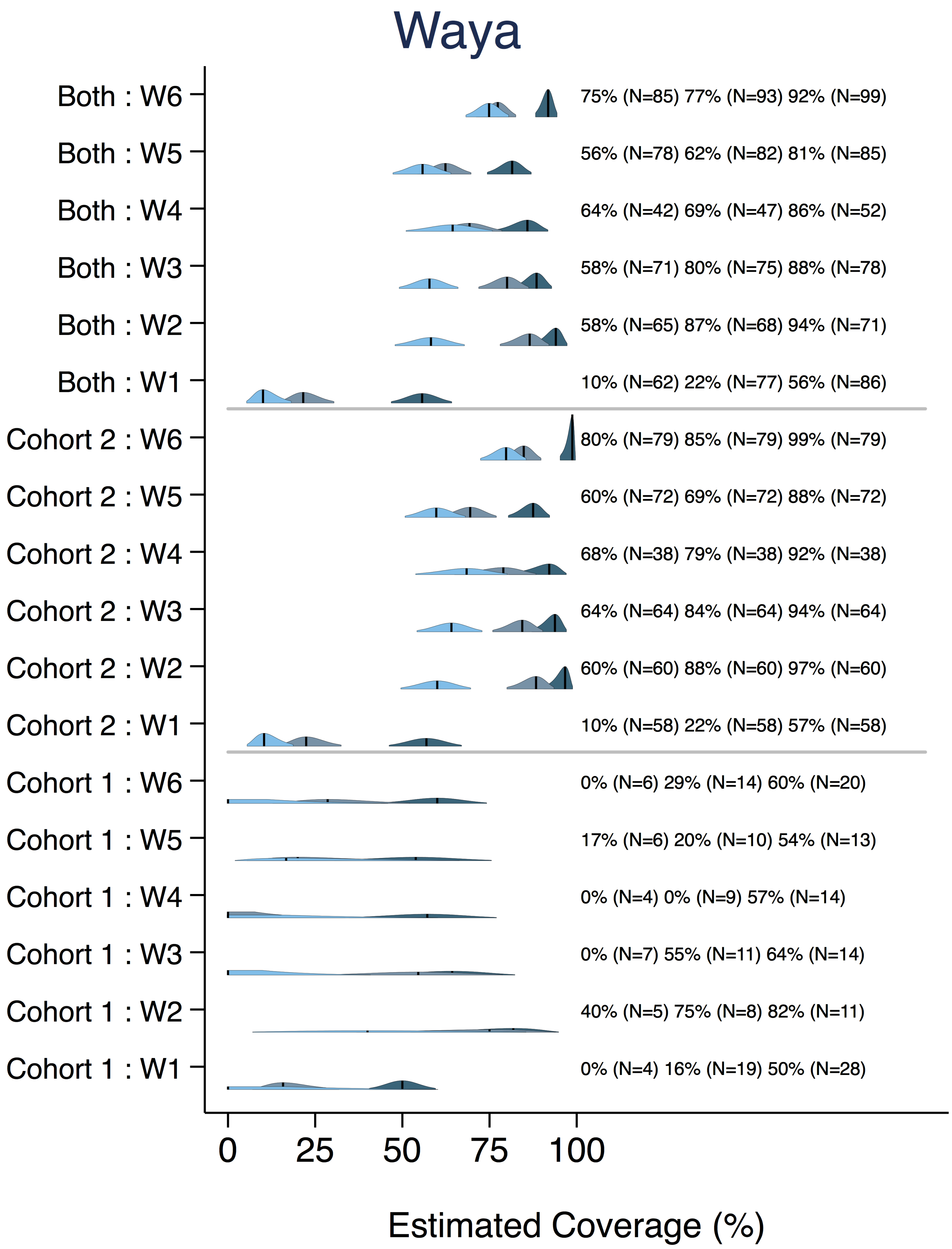

Text at right: Estimated coverge and sample size for doses 3, 2 and 1, respectively

u) Coverage in all 20 village clinic basins combined in each survey wave (W) for each age cohort (1=<4 months old; 2=4-16 months old) and both cohorts combined

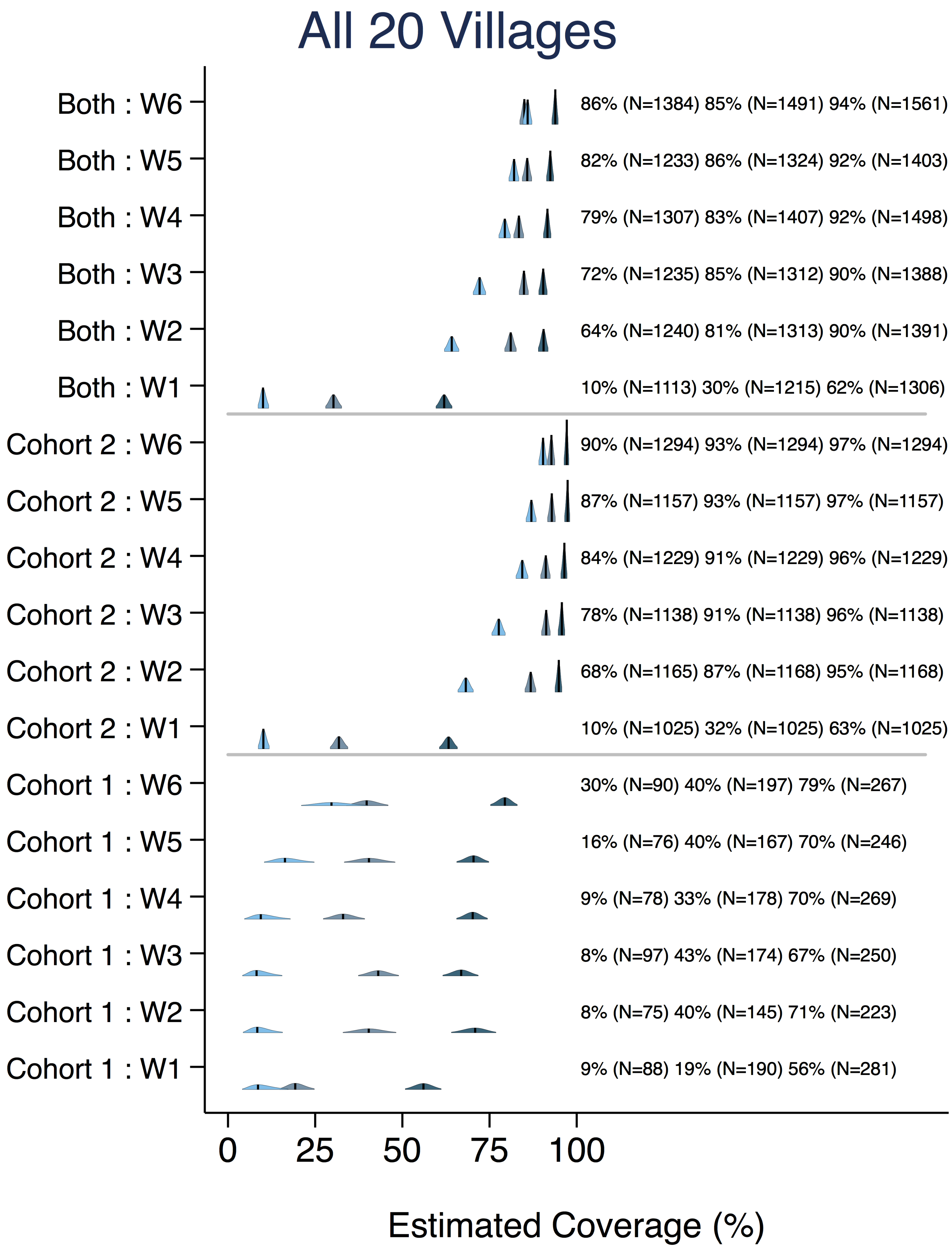

Text at right: Estimated coverge and sample size for doses 3, 2 and 1, respectively
