# Supplementary material for: An assessment of PCV13 vaccine coverage using a repeated cross-sectional household survey in Malawi
Source: Gates Open Res. 2018 Aug 2;2:37. [Version 1] doi: 10.12688/gatesopenres.12837.1 (PMC6266718; doi:10.12688/gatesopenres.12837.1)
Supplement: Peer review history

# Peer review: Reviewer response for version 1

**DOI:** 10.21956/gatesopenres.13914.r26715

**Published:** 2018-10-29

**Authors:** Stephen B. Gordon

**Affiliations:** 1 Malawi-Liverpool-Wellcome Trust Clinical Research Programme, Blantyre, Macao

**General Comments**

 This study describes PCV vaccination rates in Malawi following national implementation of the 3+0 schedule (vaccine at 6, 10 and 14 weeks of age). Malawi is a research-receptive, pro-vaccination community with a supportive Ministry of Health and a track record of success. Nevertheless, there are many operational challenges to universal vaccination owing to the very low income status of the country and the extremely stretched health services (population has quadrupled since 1970 and life expectancy increased 25% since 2000). This study is therefore welcome as it directly measures the success of an important intervention. The message that vaccination targets were not reached could reasonably be tempered with the comment that 3 vaccinations may not be necessary and the published reduction in pneumonia is striking evidence of efficacy.

**Specific Comments**

 The paper is clear and well laid out. The statistical methods are beyond a general reader. The data are presented in both tabular and graphical formats. The discussion is good and the referencing complete.

I have read this submission. I believe that I have an appropriate level of expertise to confirm that it is of an acceptable scientific standard.

# Peer review: Reviewer response for version 1

**DOI:** 10.21956/gatesopenres.13914.r26717

**Published:** 2018-10-25

**Authors:** Asnakew Tsega

**Affiliations:** 1 Maternal and Child Survival Program, Washington, DC, USA

**Overall comment**

 The survey is properly designed and the results are clear and well presented.

**Specific comments:**

**Title**

- The title looks a national coverage survey and please consider revising to indicate the specific areas. It should include **in **s**elected health districts**
- I suggest to change it to vaccination coverage than vaccine coverage.

**Methods**

- Please consider revising the definition of the **timely vaccination**, according to the Malawi immunization schedule, the age for the 1st dose of PCV13 is > 6 weeks. If the dose is administered before 6 weeks of age, it is considered as an invalid dose.
- WHO has no immunization schedule it is only recommendations to countries.
- I assume the health districts have more than one village and how were the villages identified after selecting the health district?

 
**Result**

- Vaccination campaign was mentioned a number of times in the methods, results, and discussion. The vaccine was introduced into the routine immunization system in the fixed and outreach sessions and Malawi did not do any PCV catch up vaccination campaign. At the time of introduction, all children 6 weeks to 11 months of age were eligible for the 1st dose of PCV13 and the number might have been higher than the regular vaccination, but this should not be considered as catch up, they were still in the eligible age group. 
- The availability of the health passport was very high, which is one of the strengths of the Malawi immunization program, however, there were from 2.2% to 7.6% of infants at different times without health passport. There is no explanation on how the information from the mothers' report was analyzed. How did you differentiate PCV13 from other vaccines administered at the same time, the same route and site and also if it was 1st, 2nd or 3rd dose? 

   
**Discussion**

- The spinning of a bottle is not a recommended method anymore and it would be good if you mention this as a limitation in the discussion. The recommended method is listing of all the households and randomly selecting the households for the survey.

I have read this submission. I believe that I have an appropriate level of expertise to confirm that it is of an acceptable scientific standard.

# Peer review: Reviewer response for version 1

**DOI:** 10.21956/gatesopenres.13914.r26597

**Published:** 2018-08-10

**Authors:** M. Carolina Danovaro-Holliday

**Affiliations:** 1 Expanded Programme on Immunization (EPI), Department of Immunization, Vaccines and Biologicals (IVB), World Health Organization (WHO), Geneva, Switzerland

This paper presents the results of surveys done to track uptake of pneumococcal conjugate vaccine (PCV) in rural areas in Malawi. It is well written, the results are clear, and the limitations well acknowledged. All figures are very useful, and particularly Figure 1 provides a great visual to understand survey implementation.

**Specific comments**

**Title**

- Consider adding “in selected areas in rural Malawi” to give a clear message that these surveys are not representative of the entire country

 

**Introduction**

- Pneumococcal in pneumococcal conjugate vaccines does not need to be capitalized
- The bacteria for which there is a vaccine is *Haemophilus influenzae* type b (Hib) – small b.
- In the first paragraph, consider citing the WHO Position Paper on Pneumococcus vaccines (available at: http://www.who.int/immunization/policy/position_papers/pneumococcus/en/), instead of, or in addition to, current reference 3.
- In the second paragraph, consider indicating that the target coverage rates are aligned with the Global Vaccine Action Plan (GVAP), available at: http://www.who.int/immunization/global_vaccine_action_plan/GVAP_doc_2011_2020/en/

 

**Methods**

- Include a clearer definition of “timely vaccination”. In the results, we learned what it was mainly in the footnote of Table 1.
- There is no “standard WHO Schedule” per se, see http://www.who.int/immunization/policy/immunization_tables/en/. The one used in Malawi is one of the schedules frequently used in African and other low and middle-income countries, but it is not the WHO recommended schedule per se, for example, for measles WHO recommends 9 or 12 months depending on the epidemiological situation, same for including or not an OPV0. I suggest rewording and reconsidering reference 14. It can be indicated that this is “the Malawi vaccination schedule”. To my knowledge, there is no one accepted definition of “timeliness” (see for example Tauil et al. ^1^) Furthermore, Clark and Sanderson looked at inverse surviving curves and impute dates to those without cards, which I don’t think was the case in this study.
- Under analysis, we learned that a finite population correction was used, based on an estimate of the eligible population, what is the source of this population data and year?
- Under ethical approval, were there any specific precautions taken to maintain confidentiality in relation to the pictures of health passports?

 

**Results**

- In Table 1, consider proving the availability of health passports by age group, given that it is more likely that they were available for younger infants
- In Figure 4, it is unclear who is in the denominator, all infants or those with documented dates?

 

**Discussion**

- The first paragraph talks about a “vaccination campaign”, this seems misleading as, if I understand correctly, PCV vaccine was included into routine immunization and not given using a campaign-type strategy.
- Consider adding the WHO Position Paper on Pneumococcus vaccines (available at: http://www.who.int/immunization/policy/position_papers/pneumococcus/en/), instead of, or in addition to, current reference 20.
- Is there information to describe how PCV vaccine coverage compares to coverage for other vaccines recommended at the same age (DTp-Hib-HepB, OPV)? It would be useful to better understand if PCV vaccine uptake behaves like the others or not.

I have read this submission. I believe that I have an appropriate level of expertise to confirm that it is of an acceptable scientific standard.

## References

[1] TauilMSatoAWaldmanE: Factors associated with incomplete or delayed vaccination across countries: A systematic review. *Vaccine*.2016;34(24) : 10.1016/j.vaccine.2016.04.016 2635-2643 10.1016/j.vaccine.2016.04.016 27109562
